# Leveraging the 3-Chloro-4-fluorophenyl Motif to Identify Inhibitors of Tyrosinase from *Agaricus bisporus*

**DOI:** 10.3390/ijms24097944

**Published:** 2023-04-27

**Authors:** Salvatore Mirabile, Laura Ielo, Lisa Lombardo, Federico Ricci, Rosaria Gitto, Maria Paola Germanò, Vittorio Pace, Laura De Luca

**Affiliations:** 1 Department of Chemical, Biological, Pharmaceutical and Environmental Sciences, University of Messina, Viale F. Stagno D’Alcontres 31, I-98166 Messina, Italy; salvatore.mirabile@unime.it (S.M.); lisa.lombardo@studenti.unime.it (L.L.); federico.ricci@unime.it (F.R.); rosaria.gitto@unime.it (R.G.); mariapaola.germano@unime.it (M.P.G.); 2 Foundation Prof. Antonio Imbesi, University of Messina, Piazza Pugliatti 1, I-98122 Messina, Italy; 3 Department of Chemistry, University of Turin, Via P. Giuria 7, 10125 Torino, Italy; laura.ielo@unito.it

**Keywords:** Tyrosinase, *Agaricus bisporus*, molecular modelling, 3-chloro-4-fluorophenyl moiety

## Abstract

Tyrosinase (EC 1.14.18.1) is implicated in melanin production in various organisms. There is a growing body of evidence suggesting that the overproduction of melanin might be related to several skin pigmentation disorders as well as neurodegenerative processes in Parkinson’s disease. Based on this consideration, the development of tyrosinase inhibitors represents a new challenge to identify new agents in pharmaceutical and cosmetic applications. With the goal of identifying tyrosinase inhibitors from a synthetic source, we employed a cheap and facile preliminary assay using tyrosinase from *Agaricus bisporus* (AbTYR). We have previously demonstrated that the 4-fluorobenzyl moiety might be effective in interactions with the catalytic site of AbTYR; moreover, the additional chlorine atom exerted beneficial effects in enhancing inhibitory activity. Therefore, we planned the synthesis of new small compounds in which we incorporated the 3-chloro-4-fluorophenyl fragment into distinct chemotypes that revealed the ability to establish profitable contact with the AbTYR catalytic site. Our results confirmed that the presence of this fragment is an important structural feature to improve the AbTYR inhibition in these new chemotypes as well. Furthermore, docking analysis supported the best activity of the selected studied compounds, possessing higher potency when compared with reference compounds.

## 1. Introduction

Tyrosinase (TYR, EC 1.14.18.1) is a binuclear copper containing protein expressed in various species including bacteria, fungi, plants, and animals. The main function of TYR is related to its ability to catalyze the oxidation of L-tyrosine and/or L-DOPA to furnish dopaquinone as a melanin precursor in melanosomes. Melanin plays the relevant role of a photoprotectant and is responsible for hair, skin, and eye color. However, an overproduction of melanin is considered to be related to skin disorders. Moreover, the melanin accumulation in human neurons in the substantia nigra is associated with neurodegeneration in Parkinson’s disease. The melanogenesis is a very complex and multistep process; however, the TYR activity controls the rate-limiting step, thus determining its pivotal role in the whole process of the synthesis of the physiological pigment. To date, there is a large collection of anti-melanogenic agents from synthetic [1,2,3,4] and natural sources [5,6,7,8,9]. They have been generally identified through the determination of inhibitory properties preventing the oxidation of physiological substrates (L-tyrosine or L-DOPA) in in vitro assays using enzyme from mushroom *Agaricus bisporus* (AbTYR) as a surrogate to perform a preliminary screening of potential TYR inhibitors (TYRIs). Despite there being several differences between human and mushroom isoforms, the employment of AbTYR enables a facile screening of new potential TYRIs. Interestingly, some hit compounds identified through AbTYR also demonstrated the ability to inhibit the human tyrosinase (hTYR) and produce antimelanogenic effects in human cell lines, although they proved to be less potent agents [10,11,12]. The catalytic cycle of TYR involves two distinct pathways: the hydroxylation of monophenol (monophenolase activity) and the subsequent oxidation of o-diphenol to o-quinone (diphenolase activity). From a structural point of view, the TYRs contain a catalytic pocket in which both monophenolase and diphenolase activity occurs, assisted by the two divalent copper ions surrounded by three cooperating histidine residues [13]. The various classes of reversible TYRIs exert their effects through a network of interactions with copper ions and/or specific residues paving the walls of the catalytic cavity. X-ray determination and in silico studies contributed to the elucidation of the various mechanisms of TYR inhibition as well as to the in-depth analysis of the differences between AbTYR and hTYR.

As a continuation of our previous investigations into AbTYR inhibitors, we planned the synthesis of a new small series of compounds, designed while keeping in mind that the 4-fluorobenzyl motif exerted optimized biological activity, that is, diphenolase activity of AbTYR, owing to its ability to occupy the catalytic cavity, engaging both π/π stacking and the fluorine bonding interaction [14,15,16,17,18]. In addition, it was demonstrated that an additional chlorine atom at the C-3 position of the phenyl ring provided an improvement in AbTYR inhibitory effects [19]. On the basis of these observations, we considered the 3-chloro-4-fluorophenyl moiety as an essential portion to decorate other scaffolds already distinguished as being able to engage favorable contacts within the catalytic cavity of TYRs. Herein, we report the synthesis, in vitro assay, and structure affinity relationship (SAR) considerations for this class of new compounds possessing 3-chloro-4-fluorophenyl in place of the 4-fluorobenzyl pharmacophoric feature. For selected compounds, docking simulations suggested the most plausible binding pose within the catalytic site of TYRs.

## 2. Results

### 2.1. Lead Optimization Strategy

**Figure 1:**
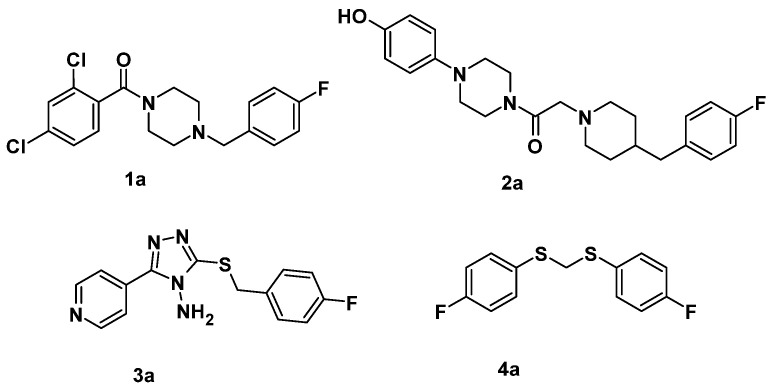
Chemical structures of selected AbTYR inhibitors **1a**, **2a**, **3a**, and **4a** bearing the 4-fluorophenyl pharmacophoric feature.

The first goal of our hit optimization strategy was the introduction of the 3-chloro-4-fluorophenyl moiety in the aromatic tail of several piperazine-based compounds, such as 1-(2,4-dichlorobenzoyl)-4-[(4-fluorophenyl)methyl]piperazine (**1a**) [18] and 2-(4-[(4-fluorophenyl)methyl]piperidin-1-yl)-1-[4-(4-hydroxyphenyl)piperazin-1-yl]ethan-1-one (**2a**) [20], that have been identified as inhibitors toward AbTYR (see Figure 1) at a low micromolar concentration. Based on the consideration that the replacement of the cyclic amine core with a 5-(pyridin-4-yl)-3-(alkylsulfanyl)-4*H*-1,2,4-triazol-4-amine moiety resulted a further series of AbTYR inhibitors (e.g., 3-([(4-fluorophenyl)methyl]sulfanyl)-5-(pyridin-4-yl)-4*H*-1,2,4-triazol-4-amine, **3a**) [14], we chose to introduce a few structural modifications in this class of compounds as well. Finally, we explored in depth a further series of potential inhibitors, starting from the active 1,1’-[methylenebis(sulfanediyl)]bis(4-fluorobenzene) (**4a**) as an AbTYR inhibitor [16], which we have previously identified by means of a virtual screening campaign on a database of naturally inspired compounds.

### 2.2. In Vitro Assay Determination of AbTYR Inhibition

Our optimization strategy led to the design of sixteen new derivatives (Table 1). To assess the inhibition of AbTYR of new compounds, we employed the method previously described for parent compounds **1a**, **2a**, **3a**, and **4a**, with a few modifications. Table 1 collects the IC_50_ values of inhibitory effects for the new designed compounds **1d–f**, **2c–d**, **3b**, **3d**, and **4d–f** bearing the 3-chloro-4-fluorophenyl fragment, in comparison with their parent compounds **1a–c** [15,17,18], **2a** [20], **3a** [14], and **4a** [16], which have already been reported, as well as new analogue derivatives **2b**, **3c**, **4b–c**, **4g**, and **4h**, which were synthesized for comparison purposes. All data were compared to the activity of kojic acid (KA), which is considered the canonical reference compound in this screening protocol [15].

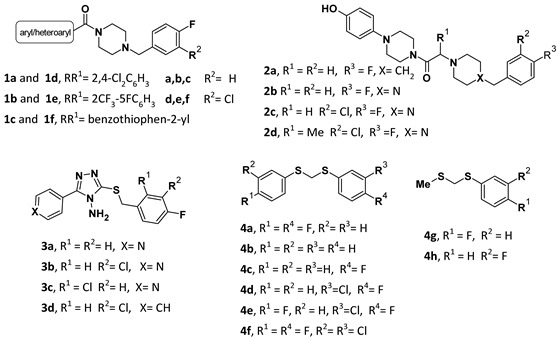



**Table 1:** Biochemical data of diphenolase inhibition of AbTYR for compounds **1a–f**, **2a–d**, **3a–d**, and **4a–h**, as well as the reference compound kojic acid (KA).

Entry	IC_50_ (μM) ^a^ ± SD ^b^
**1a ^c^**	0.79 ± 0.11
**1b ^d^**	0.24 ± 0.03
**1c ^e^**	3.60 ± 0.33
**1d**	0.42 ± 0.03
**1e**	0.19 ± 0.07
**1f**	1.72 ± 0.11
**2a ^f^**	4.49 ± 0.13
**2b**	4.43 ± 0.54
**2c**	1.73 ± 0.28
**2d**	1.38 ± 0.15
**3a ^g^**	83.61 ± 15.65
**3b**	24.15 ± 1.02
**3c**	>350
**3d**	67.32 ± 6.67
**4a ^h^**	48.42 ± 0.76
**4b**	>350
**4c**	>350
**4d**	6.26 ± 0.37
**4e**	10.65 ± 1.51
**4f**	2.96 ± 0.34
**4g**	26.02 ± 2.63
**4h**	183.46 ± 6.12
Kojic Acid (KA) ^c^	17.76 ± 0.18

^a^ All compounds were tested using a set of experiments performed in triplicate; IC_50_ values correspond to the concentration causing 50% enzyme activity loss. ^b^ SD represents the standard deviation. ^c^ Data taken from [18]. ^d^ Data taken from [15]. ^e^ Data taken from [17]. ^f^ Data taken from [20]. ^g^ Data taken from [14]. ^h^ Data taken from [16].

The new 3-chloro-4-fluorophenyl-based benzamide compounds **1d–f** achieved significant IC_50_ values, spanning from 0.19 to 1.72 μM, as AbTYR inhibitors, showing a light improvement in potency with respect to previous reports for active 4-fluorophenyl-based analogues **1a–c**. Based on the confirmation of the activity of compounds containing the 3-chloro-4-fluorophenyl moiety, we further adopted this strategy toward 4-hydroxyphenyl-based compounds and studied new analogues of prototype **2a** (IC_50_ value of 4.49 μM). Considering that the piperazine core could be more prone to being variously decorated, our investigation began with the evaluation of AbTYR inhibitory effects of analogue compound **2b**, which was demonstrated to still be an active inhibitor (IC_50_ value of 4.43 μM). Subsequently, we introduced the additional 3-chloro-4-fluorosubstituent to furnish the active compound **2c** (IC_50_ value of 1.73 μM); when a methyl group was added to the acetyl linking group, we obtained the homologue compound **2d** (IC_50_ value of 1.38 μM), which displayed a similar potency to compound **2c**, suggesting that the methyl group occupied a large area of the pocket and that the change in steric hindrance of the linking group did not affect the binding interaction. The chlorine atom was also introduced on the 3-position of the phenyl ring of the previously reported 5-(pyridin-4-yl)-3-(alkylsulfanyl)-4*H*-1,2,4-triazol-4-amine-based derivative **3a**; inhibitor **3b** was obtained, which was about 3.5-fold more potent than parent compound **3a**. In contrast, the inhibitory effects dramatically disappeared when the chlorine atom was positioned at the 2-position of the phenyl ring, as evidenced for derivative **3c**. To gain further information, we replaced the pyridin-4-yl-substituent with the phenyl ring to furnish compound **3d**; as a result, the AbTYR inhibitory activity was lower than that of parent compound **3b**. Finally, we extended our investigations toward a series of alkylsulfanyl-based compounds **4b–h**; these new potential AbTYR inhibitors were inspired by prototype **4a**, which emerged from an in silico screening campaign in our previous studies. In the current investigation, we introduced a few structural modifications and replaced the aromatic feature with small alkyl groups. Among the series of compounds **4b–f** bearing two aromatic tails, it might be observed that the presence of the 3-chloro-4-fluorophenyl fragment led to a remarkable enhancement in the inhibitory activity; the unsubstituted compounds **4b–c** were inactive agents, whereas the poly-substituted derivatives **4d–f** displayed inhibitory effects in the low micromolar range (IC_50_ values from 2.96 to 10.65 μM); they were still more potent than the precursor **4a**. By comparing the IC_50_ values of the two methylsulfanyl-derivatives **4g** and **4h**, we confirmed that the 4-fluorine atom substitution gained better AbTYR affinity than that of the 3-fluorine atom substitution.

### 2.3. Docking Studies on Mushroom Tyrosinase

**Figure 2:**
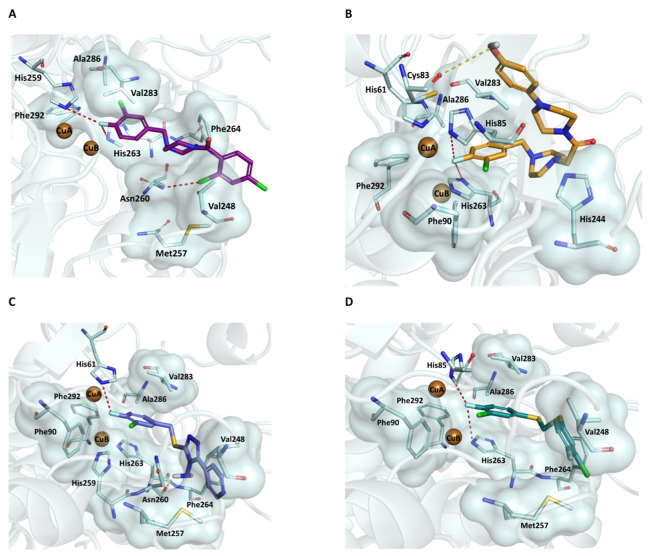
The best poses of the compounds **1d** (purple sticks) (**A**), **2c** (orange sticks) (**B**), **3b** (slate sticks) (**C**), and **4f** (deep teal sticks) (**D**) docked in the catalytic cavity, where the di-copper ions are represented as brown spheres. The residues involved in the interactions are depicted as pale cyan sticks, adding the surface in the case of hydrophobic contacts. Halogen and hydrogen bonds are highlighted as red and yellow dashes, respectively. The AbTYR is depicted as a cartoon. The figure was generated using PyMOL software (www.pymol.org).

To confirm the important role of the 3-chloro-4-fluorophenyl fragment in the binding to the AbTYR catalytic pocket, a semi-flexible docking protocol was applied for selected representative compounds **1d**, **2c**, **3b**, and **4f** using the software Gold [21]. The results of our molecular modelling analysis of the best potent inhibitors **1d**, **2c**, **3b**, and **4f** are depicted in Figure 2. For all of the docked inhibitors, the fluorine atom formed relevant contacts in the AbTYR cavity: (i) it was involved in halogen bond interactions with the copper-coordinating histidine; (ii) it generally acted as a metal acceptor toward di-copper ions CuA and CuB, with the exception of the compound **3b**, which interacted exclusively with CuA (see Figure 2C); and (iii) it formed hydrophobic contacts with Phe292. Furthermore, we observed that the 4-fluorosubstituted phenyl ring interacted by π–π stacks with the side chain of His263. Particularly, in compounds **1d**, **3b**, and **4f**, the different fragments characterizing each chemotype showed a similar rearrangement, engaging favorable contacts with the residues forming the entrance of the catalytic site, such as Val248, Met257, Asn260, and Phe264.

A distinguishable binding mode is observed for compound **2c**, probably due to its greater length compared with the other candidates. As a result, folding of the 4-piperazine-phenyl tail occurs to accommodate the ligand in the pocket, stabilized by hydrophobic contacts with the side chain of His244 and Val283 and a hydrogen bond between the phenolic OH group and Cys83, supporting that the increase in chain length should not affect the binding affinity.

Moreover, despite that a different fitting of 3-chloro-4-fluorophenyl is encountered in all compounds, the chlorine atom stabilized the binding to the catalytic cavity through van der Waals interactions, specifically with Ala286 and Val283 in the ligand **1d** and with Phe90 and Val283 in the inhibitors **2c**, **3b**, and **4f**. The collected results corroborated the importance of this moiety and were consistent with the biochemical data displayed in Table 1.

### 2.4. Chemistry

**Scheme 1:**
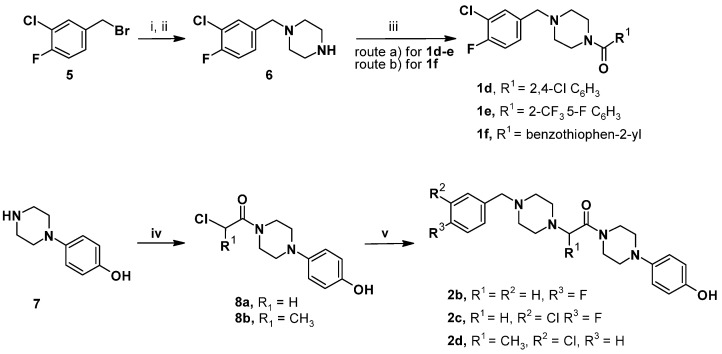
Synthesis of the designed compounds **1d–f** [18] and **2b–d** [20]. Reagents and conditions: (i) *tert*-butylpiperazine-1-carboxylate, K_2_CO_3_, EtOH, reflux, 18 h, 98 %; (ii) TFA, dichloromethane (DCM), rt, 5 h, ca 60% over two steps; (iii) route (a) RCOCl, *N,N*-diisopropylethylamine (DIPEA), DCM, rt, 5 h, 40–58%, and route (b) RCOOH, triethylamine (TEA), *N,N,N′,N′*-tetramethyl-O-(1*H*-benzotriazol-1-yl)uronium hexafluorophosphate (HBTU), *N,N*-dimethylformamide (DMF), rt, 18 h, 51%; (iv) ClCOCH(R_1_)Cl, DMF, rt, 3 h, 49–64%; (v) 1-[(4-fluorophenyl)methyl]piperazine (**9**) or [(3-chloro-4-fluorophenyl)methyl]piperazine (**6**), K_2_CO_3_, DMF, reflux, 18 h, 39–85%.

**Scheme 2:**
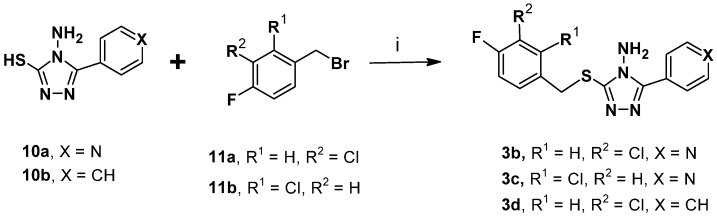
Synthesis of the designed compounds **3b–d** [14]. Reagents and conditions: (i) NaOH, MeOH, rt, 3 h, quantitative.

**Scheme 3:**
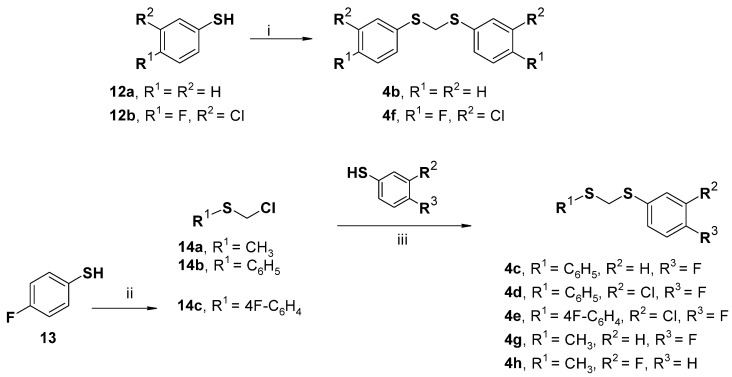
Synthesis of the designed compounds **4b–h**. Reagents and conditions: (i) NaOH, CH_2_I_2_, *i*PrOH, 80 °C, 2 h, 93% [22]; (ii) preparation of starting material **14c**: paraformaldehyde, toluene/HCl, 50 °C 2 h to rt 3 h, 90% [23]; (iii) K_2_CO_3_, DMF, rt, overnight, 87–94% [24].

The synthesis of the designed compounds was performed employing the synthetic routes described in Scheme 1, Scheme 2 and Scheme 3. The preparation of the designed compounds followed the procedures described in our previous publications [14,18,20], with slight modifications.

The first series of three target compounds **1d–f** was readily prepared in three steps in a moderate yield via the *N*-alkylation reaction, removal of the protective group, and condensation with carboxylic acid or acyl-chlorides, as reported in Scheme 1. In detail, the key intermediate 1-[(3-chloro-4-fluorophenyl)methyl]piperazine (**6**) was generated by the reaction of 3-chloro-4-fluorobenzylbromide (**5**) with *tert*-butylpiperazine-1-carboxylate and the subsequent deprotection with trifluoroacetic acid (TFA) in dichloromethane (DCM). The intermediate **6** reacted with aryl/heteroaryl-compounds to generate variously substituted piperazine-amides **1d–f** under distinct experimental conditions (see Scheme 1). Meanwhile, the desired compounds **2c–d** were prepared in a two-step sequence in moderate-to-good yields through the coupling reaction of 4-hydroxyphenylpiperazine (**7**) with 2-chloroacetyl chloride or 2-chloropropionyl chloride at room temperature in *N,N*-dimethylformamide (DMF) to yield the intermediate **8a** or **8b**, and subsequent reaction with 1-[(3-chloro-4-fluorophenyl)methyl]piperazine **6** as the central reagent to obtain this series of compounds **2c** and **2d**, respectively. The parent 4-fluoro-substituted compound **2b** was prepared under similar conditions using 1-[(4-fluorophenyl)methyl]piperazine (**9**) in place of 1-[(3-chloro-4-fluorophenyl)methyl]piperazine (**6**).

The synthesis of the compounds **3b–d** bearing the 4*H*-1,2,4-triazol-4-amine core was carried out by the coupling of reactants **10a–b** with the suitable benzylbromide **11a–b** under basic conditions, as displayed in Scheme 2.

Moreover, we prepared compounds **4b–h** displaying an alternative fragment to incorporate the pharmacophoric 3-chloro-4-fluorophenyl moiety. To obtain the targeted compounds, the synthetic procedure described in Scheme 3 was applied, thus furnishing the desired compounds in a good yield. In detail, derivatives **4b** and **4f** were synthesized by reacting the suitable thiophenol (**12a–b**) with diiodomethane and NaOH. Compounds **4c–e** and **4g–h** were prepared via a simple nucleophilic substitution between the proper thiophenol and the chloro(methylsulfanyl) derivative (**14a–c**) in a basic environment. The starting materials chloro(methylsulfanyl)methane (**14a**) and [(chloromethyl)sulfanyl]benzene (**14b**) were commercially available, whereas 1-[(chloromethyl)sulfanyl]-4-fluorobenzene (**14c**) was prepared by the reaction of 4-fluorobenzene-1-thiol (**13**) with paraformaldehyde.

All compounds were structurally characterized through spectroscopic measurements, as reported in Section 3 and the Supplementary Materials.

## 3. Materials and Methods

### 3.1. Biochemical Assays

Mushroom tyrosinase (EC 1.14.18.1) was supplied by Merck (Cat. No. T3824). In vitro assay was performed according to the method of Mirabile et al. [15]. Each sample (0.05 mL) at different concentrations (0.10–200 μM) was mixed with 0.5 mL of L-DOPA solution (1.25 mM) and 0.9 mL of phosphate buffer solution (pH 6.8). After a 10 min incubation at 25 °C, 0.05 mL of an aqueous solution of the enzyme was added to the mixture. Absorbance was recorded at 475 nm using a spectrophotometer (Agilent Technologies, Cary 60, UV/Vis). Kojic acid [5-hydroxy-2-(hydroxymethyl)-4-pyran-4-one] was employed as a positive standard (10–30 μM). The inhibition rate was calculated with the following mathematical equation:

Inhibition % = (A − B/A) × 100

where A = absorbance of negative control and B = absorbance of test sample.

Finally, IC_50_ values were determined by interpolation of the dose–response curves.

### 3.2. Molecular Modelling

Docking studies were carried out using Gold software V 2020.2.0 [21]. To achieve this purpose, our studies were carried out on the basis of the structure of the complex of *Agaricus bisporus tyrosinase* (AbTYR) and tropolone (PDB ID 2Y9X) [25]. The protocols applied for the protein and ligands’ preparation and the docking calculation have been reported in our previous works [18,20]. The analysis of the docked poses was carried out by means Discovery Studio Visualizer [26] and Ligand Scout V 4.4.9 [27].

### 3.3. Chemistry

All of the employed reagents were purchased from common commercial suppliers (Sigma-Aldrich, Milan, Italy and Alfa Aesar, Karlsruhe, Germany). A Buchi B-545 instrument (BUCHI Labortechnik AG, Flawil, Switzerland) was used to determine melting points. Combustion analysis (C, H, N), performed with a Carlo Erba Model 1106-Elemental Analyzer (Milan, Italy), revealed ≥95% purity of the obtained compounds. Merck Silica Gel 60 F254 plates (Milan, Italy) were employed for thin-layer chromatography (TLC; Merck KGaA, Darmstadt, Germany). ^1^H NMR and ^13^C NMR spectra were recorded in deuterated dimethylsulfoxide (DMSO-*d_6_*) or chloroform (CDCl_3_) with a Varian Gemini 500 spectrometer (Palo Alto, CA, USA), Bruker Avance III 400 (Rheinstetten, Germany), or Jeol ECZR600 (Milan, Italy), with chemical shifts (δ) expressed in ppm and coupling constants (*J*) in hertz (Hz).

#### 3.3.1. General Procedures for the Synthesis of Key Intermediate 1-[(3-Chloro-4-fluorophenyl)methyl]piperazine (**6**)

A mixture of *tert*-butyl piperazine-1-carboxylate (300 mg, 1.61 mmol), 3-chloro-4-fluorobenzylbromide (5, 217.6 µL, 1.61 mmol), and K_2_CO_3_ (445 mg, 3.22 mmol) in EtOH (10 mL) was refluxed for 18 h. Then, the mixture was diluted with water (20 mL) and extracted with DCM (20 mL, ×3) to furnish different organic phases, which were collected and dried over Na_2_SO_4_; then, the mixture was concentrated in vacuo to yield the intermediate *tert*-butyl 4-[(3-chloro-4-fluorophenyl)methyl]piperazine-1-carboxylate. Subsequently, a solution of *tert*-butyl 4-[(3-chloro-4-fluorophenyl)methyl]piperazine-1-carboxylate (519 mg, 1.57 mmol) in DCM (8 mL) was treated with TFA (15.7 mmol); the resulting mixture was stirred at room temperature for 5 h. After the reaction was completed, it was cooled on ice and diluted with DCM (2 mL) and a solution of K_2_CO_3_ (2M, 2 mL). The mixture was extracted with DCM (10 mL, ×2); the combined organic layers were dried over Na_2_SO_4_ and the solvent was removed in vacuo. Finally, the desired intermediate 1-[(3-chloro-4-fluorophenyl)methyl]piperazine (**6**) was obtained by crystallization with diethyl ether.

*tert*-Butyl 4-[(3-chloro-4-fluorophenyl)methyl]piperazine-1-carboxylate (intermediate)

Yield: 98%. Oily residue. ^1^H-NMR (500 MHz, CDCl_3_): (δ) 1.44 (s, 9H, 3CH_3_), 2.35 (bs, 4H, 2CH_2_), 3.40 (m, 4H, 2CH_2_), 3.42 (s, 2H, CH_2_), 7.06 (t, *J* = 8.7 Hz, 1H, ArH, H-5′), 7.16 (m, 1H, ArH, H-6′), 7.36 (d, *J* = 7.1 Hz, 1H, ArH, H-2′). Anal. Calculated for C_16_H_22_ClFN_2_O_2_: C 58.44, H 6.74, N 8.52. Found: C 58.39, H 6.71, N 8.56.

1-[(3-Chloro-4-fluorophenyl)methyl]piperazine (**6**)

Yield: 62%. White powder. M.p. 147–150 °C. ^1^H-NMR (500 MHz, DMSO-d6): (δ) 2.54 (m, 4H, 2CH_2_), 3.08 (t, *J* = 5.1 Hz, 4H, 2CH_2_), 3.33 (s, 1H, NH), 3.53 (s, 2H, CH_2_), 7.33 (m, 1H, ArH, H-5′), 7.39 (t, *J* = 8.8 Hz, 1H, ArH, H-6′), 7.53 (dd, *J* = 7.4, 2.1 Hz, 1H, ArH, H-2′). Anal. Calculated for C_11_H_14_ClFN_2_: C 57.77, H 6.17, N 12.25. Found: C 57.81, H 6.13, N 12.19.

#### 3.3.2. General Procedures for the Synthesis of Amides **1d–f**

Route (a) To a solution of 1-[(3-chloro-4-fluorophenyl)methyl]piperazine (**6**) (0.66 mmol) in DCM (4 mL), the suitable benzoyl chloride (0.66 mmol) and *N,N*-diisopropylethylamine (DIPEA, 0.99 mmol) were added. The reaction mixture was stirred at room temperature for 5 h; after the completion of the reaction, the work-up to obtain target compounds **1d** and **1e** was performed as previously reported for analogues **1a** and **1b** [15,18].

Route (b) A mixture of benzothiophene-2-carboxylic acid (1.05 mmol) and *N,N,N′,N′-*tetramethyl-O-(1*H*-benzotriazol-1-yl)uronium hexafluorophosphate (HBTU) (1.05 mmol) in DMF (4 mL) was stirred at room temperature for 1 h. Then, 1-[(3-chloro-4-fluorophenyl)methyl]piperazine (1.05 mmol) and TEA (2.1 mmol) were added and it was stirred for 17 h. The target compound **1f** was obtained as previously reported for analog compound **1c** [17].

(4-(3-Chloro-4-fluorobenzyl)piperazin-1-yl)(2,4-dichlorophenyl)methanone (**1d**)

Yield: 58%. White powder. M.p. 121–124 °C. ^1^H-NMR (500 MHz, DMSO-*d*_6_): (δ) 2.33 (t, *J* = 3.36 Hz, 2H, CH_2_), 2.43 (m, 2H, CH_2_), 3.13 (m, 2H, CH_2_), 3.49 (s, 2H, CH_2_), 3.63 (m, 2H, CH_2_), 7.31 (m, 1H, ArH, H-5′), 7.35 (d, *J* = 9.2 Hz, 1H, ArH, H-6′), 7.39 (m, 1H, ArH, H-2′), 7.49 (d, *J* = 2.02 Hz, 1H, ArH, H-5”), 7.51 (d, *J* = 2.02 Hz, 1H, ArH, H-6”), 7.72 (d, *J* = 1.9 Hz, 2H, ArH, H-3”). ^13^C-NMR (126 MHz, DMSO-d6): (δ) 41.1 (C-5), 46.2 (C-3), 51.8 (C-2), 52.5 (C-6), 60.1 (CH_2_), 116.6 (d, *J_C-F_* = 20.77 Hz, C-5′), 119.1 (d, *J_C-F_* = 17.66 Hz, C-3′), 127.9 (C-5”), 129.1 (C-6′), 129.3 (C-6”), 129.4 (C-3″), 130.3 (C-2′), 130.6 (C-2″), 134.2 (C-4″), 134.7 (C-1″), 135.9 (d, *J_C-F_* = 3.86 Hz, C-1′), 156.3 (d, *J_C-F_* = 245.5 Hz, C-4′), 164.6 (C=O). Anal. Calculated for C_18_H_16_Cl_3_FN_2_O: C 53.82, H 4.01, N 6.97. Found: C 53.88, H 3.99, N 7.03.

(4-(3-Chloro-4-fluorobenzyl)piperazin-1-yl)(5-fluoro-2-(trifluoromethyl)phenyl)methanone (**1e**)

Yield: 40%. White powder. M.p. 133–135 °C. ^1^H-NMR (500 MHz, CDCl_3_): (δ) 2.42 (bs, 2H, CH_2_), 2.59 (bs, 2H, CH_2_), 3.24 (bs, 2H, CH_2_), 3.55 (s, 2H, CH_2_), 3.84 (m, 2H, CH_2_), 7.03 (d, *J* = 8.1 Hz, 1H, ArH, H-5′), 7.09 (m, 1H, ArH, H-6′), 7.20 (m, 2H, ArH, H-3″ and H-6″), 7.39 (m, 1H, ArH, H-2′), 7.70 (m, 1H, ArH, H-4″). ^13^C-NMR (126 MHz, CDCl_3_): (δ) 41.6 (C-5), 46.9 (C-3), 52.3 (C-2), 52.4 (C-6), 61.4 (CH_2_), 114.9 (d, *J_C-F_* = 23.67 Hz, C-5′), 116.5 (d, *J_C-F_* = 21.86 Hz, C-6″), 116.6 (d, *J_C-F_* = 21.05 Hz, C-4″), 116.9 (C-2″), 121.1 (q, *J_C-F_* = 17.8 Hz, CF_3_, C-3′), 124.4 (d, *J_C-F_* = 272.45 Hz, CF_3_), 128.9 (d, *J_C-F_* = 7.2 Hz, C-6′), 129.6 (q, *J_C-F_* = 4.5 Hz, C-3″), 131.2 (C-2′), 134.1 (C-1′), 137.4 (C-1″), 157.6 (d, *J_C-F_* = 248.6 Hz, C-4′), 164.4 (d, *J_C-F_* = 255.5 Hz, C-5″), 165.9 (C=O). Anal. Calculated for C_19_H_16_ClF_5_N_2_O: C 54.49, H 3.85, N 6.69. Found: C 54.56, H 3.90, N 6.62.

Benzo[*b*]thiophen-2-yl(4-(3-chloro-4-fluorobenzyl)piperazin-1-yl)methanone (**1f**)

Yield: 51%. White powder. M.p. 111–113 °C. ^1^H-NMR (500 MHz, CDCl_3_): (δ) 2.49 (bs, 4H, 2CH_2_), 3.49 (s, 2H, CH_2_), 3.79 (bs, 4H, 2CH_2_), 7.09 (m, 1H, ArH, H-5′), 7.18 (m, 1H, ArH, H-6′), 7.39 (m, 3H, ArH, H-2″, H-5″ and H-6″), 7.46 (bs, 1H, ArH, H-2′), 7.80 (m, 1H, ArH, H-4″), 7.85 (m, 1H, ArH, H-5″). ^13^C-NMR (126 MHz, CDCl_3_): (δ) 29.8 (C-3 and C-5), 53.1 (C-2 and C-6), 61.7 (CH_2_), 116.5 (d, *J_C-F_* = 20.95 Hz, C-5′), 121.0 (C-3′), 122.5 (C-3″), 124.7 (C-7″), 124.9 (C-3″a), 125.3 (C-5″) 125.9 (C-6″), 128.6 (d, *J_C-F_* = 7.05 Hz C-6′), 131.0 (C-2′), 134.9 (d, *J_C-F_* = 3.74 Hz C-1′), 136.7 (C-3″), 138.7 (C-7″a) 140.3 (C-2″), 157.5 (d, *J_C-F_* = 247.4 Hz, C-4′), 163.9 (C=O). Anal. Calculated for C_20_H_18_ClFN_2_OS: C 61.77, H 4.67, N 7.20. Found: C 61.80, H 6.64, N 7.22.

#### 3.3.3. General Procedures for the Synthesis of Amides **2b–d**

To a solution of 4-(1-piperazinyl)phenol (7, 300 mg, 1.68 mmol) in DMF (4 mL), the suitable acyl chloride (1.68 mmol) was slowly added at 0 °C. Then, the reaction mixture was stirred at room temperature for 3 h. After the completion of the reaction, as indicated by TLC, a saturated solution of NaHCO_3_ (5 mL) was added to quench the reaction. The reaction mixture was extracted with EtOAc (×2); the organic layers were collected and dried over Na_2_SO_4_ and the solvent was removed under vacuum to afford intermediates 2-chloro-1-[4-(4-hydroxyphenyl)piperazin-1-yl]ethanone (**8a**, R^1^ = H) or 2-chloro-1-(4-(4-hydroxyphenyl)piperazin-1-yl)propan-1-one (**8b**, R^1^ = Me) as powder through treatment with EtOH and Et_2_O. To a solution of 2-chloro-1-[4-(4-hydroxyphenyl)piperazin-1-yl]ethanone (**8a**, 210 mg, 0.82 mmol) or 2-chloro-1-(4-(4-hydroxyphenyl)piperazin-1-yl)propan-1-one (**8b**, 220 mg, 0.82 mmol) in DMF (4 mL), 1-[(3-chloro-4-fluorophenyl)methyl]piperazine (**6**) (0.82 mmol) or 1-[(4-fluorophenyl)methyl]piperazine (**9**, 0.82 mmol) was added. This was followed by the addition of K_2_CO_3_ (56.7 mg, 0.41 mmol); then, the reaction mixture was refluxed for 18 h and quenched with a saturated solution of NaHCO_3_ (5 mL). The aqueous layer was extracted with EtOAc (3 × 10 mL) and the obtained organic phases were washed with brine, dried over Na_2_SO_4_, filtered, and concentrated under a reduced pressure. Finally, the desired compounds **2b–d** were crystallized with Et_2_O and EtOH.

2-Chloro-1-[4-(4-hydroxyphenyl)piperazin-1-yl]ethanone (**8a**)

Yield: 49%. White powder. M.p.: 162–163 °C. ^1^H-NMR (500 MHz, DMSO-*d_6_*): (δ) 2.92 (m, 2H, CH_2_), 2.98 (m, 2H, CH_2_), 3.58 (m, 4H, 2CH_2_), 4.41 (s, 2H, CH_2_), 6.66 (d, *J* = 8.9 Hz, 2H, ArH, H-2′ and H-6′), 6.81 (d, *J* = 8.9 Hz, 2H, ArH, H-3′ and H-5′), 8.89 (bs, 1H, OH). Anal. Calculated for C_12_H_15_ClN_2_O_2_: C 56.59, H 5.94, N 11.00. Found: C 56.65, H 5.83, N 11.07. The structural characterization of the pure intermediate 2-chloro-1-[4-(4-hydroxyphenyl)piperazin-1-yl]ethanone (**8a**) was in good agreement with previous data reported in the literature [20].

2-Chloro-1-(4-(4-hydroxyphenyl)piperazin-1-yl)propan-1-one (**8b**)

Yield: 64%. White powder. M.p.: 148–150 °C. ^1^H-NMR (500 MHz, DMSO-*d_6_*): (δ) 1.52 (d, *J* = 6.4 Hz, 3H, CH_3_), 2.96 (m, 4H, 2CH_2_), 3.63 (m, 4H, 2CH_2_), 5.09 (q, *J* = 6.4 Hz, 1H, CH), 6.66 (d, *J* = 8.9 Hz, 2H, ArH, H-2′ and H-6′), 6.81 (d, *J* = 8.9 Hz, 2H, ArH, H-3′ and H-5′), 8.88 (bs, 1H, OH). Anal. Calculated for C_13_H_17_ClN_2_O_2_: C 58.10, H 6.38, N 10.42. Found: C 58.16, H 6.40, N 10.39.

2-(4-(4-Fluorobenzyl)piperazin-1-yl)-1-(4-(4-hydroxyphenyl)piperazin-1-yl)ethanone (**2b**)

Yield: 85%. White powder. M.p. 185–187 °C. ^1^H-NMR (500 MHz, DMSO-*d*_6_): (δ) 2.38 (m, 6H, 3CH_2_), 2.87 (bs, 2H, CH_2_), 2.95 (bs, 2H, CH_2_), 3.15 (s, 2H, CH_2_), 3.22 (s, 2H, CH_2_), 3.42 (s, 2H, CH_2_), 3.55 (bs, 2H, CH_2_), 3.65 (bs, 2H, CH_2_), 6.66 (d, *J* = 8.84 Hz, 2H, ArH, H-2′ and H-6′), 6.80 (d, *J* = 8.84 Hz, 2H, ArH, H-3′ and H-3′), 7.12 (m, 2H, ArH, H-3″ and H-5″), 7.30 (m, 2H, ArH, H-2″ and H-6″), 8.86 (s, 1H, OH). ^13^C-NMR (126 MHz, DMSO-*d*_6_): (δ) 41.2 (C-2a and C-6a), 45.3 (C-3a and C-5a), 50.3 (C-3b and C-5b), 50.9 (C-2b and C-6b), 52.5 (CH_2_-C=0), 61.1 (CH_2_), 114.8 (d, *J_C-F_* = 20.84 Hz, C-3″ and C-5″), 115.4 (C-2′ and C-6′), 118.3 (C-3′ and C-5′), 130.6 (d, *J_C-F_* = 7.82 Hz, C-2″ and C-6″), 134.3 (C-1″), 144.0 (C-1′), 151.3 (C-4′), 161.2 (d, *J_C-F_* = 241.71 Hz, C-4″), 167.3 (C=O). Anal. Calculated for C_23_H_29_FN_4_O_2_: C 66.97, H 7.09, N 13.58. Found: C 67.00, H 7.12, N 13.54.

2-(4-(3-Chloro-4-fluorobenzyl)piperazin-1-yl)-1-(4-(4-hydroxyphenyl)piperazin-1-yl)ethanone (**2c**)

Yield: 53%. White powder. M.p. 165–168 °C. ^1^H-NMR (500 MHz, DMSO-*d*_6_): (δ) 2.40 (m, 4H, 2CH_2_), 2.88 (m, 2H, CH_2_), 2.96 (m, 2H, CH_2_), 3.22 (m, 2H, CH_2_), 3.46 (s, 2H, CH_2_), 3.56 (m, 2H, CH_2_), 3.64 (s, 2H, CH_2_), 6.66 (d, *J* = 8.94 Hz, 2H, ArH, H-2′ and H-6′), 6.80 (d, *J* = 8.94 Hz, 2H, ArH, H-3′ and H-3′), 7.30 (m, 1H, ArH, H-5″), 7.35 (t, *J* = 6.6 Hz, 1H, ArH, H-6″), 7.48 (d, *J* = 8.9 Hz, 1H, ArH, H-2″), 8.88 (s, 1H, OH). ^13^C-NMR (126 MHz, DMSO-*d*_6_): (δ) 41.3 (C-2a and C-6a), 45.2 (C-3a and C-5a), 50.3 (C-3b and C-5b), 50.9 (C-2b and C-6b), 52.4 (CH_2_-C=0), 60.2 (CH_2_), 115.5 (C-2′ and C-6′), 116.6 (C-5″), 118.4 (C-3′ and C-5′), 118.5 (C-3″), 119.1 (C-6″), 129.3 (C-2″), 130.6 (C-1″), 143.9 (C-1′), 151.3 (C-4′), 156.2 (d, *J_C-F_* = 245.23 Hz, C-4″), 172.0 (C=O). Anal. Calculated for C_23_H_28_ClFN_4_O_2_: C 61.81, H 6.31, N 12.54. Found: C 61.84, H 6.35, N 12.50.

2-(4-(3-Chloro-4-fluorobenzyl)piperazin-1-yl)-1-(4-(4-hydroxyphenyl)piperazin-1-yl)propan-1-one (**2d**)

Yield: 39%. Beige powder. M.p. 172–174 °C. ^1^H-NMR (500 MHz, DMSO-*d*_6_): (δ) 1.03 (d, *J* = 6.64 Hz, 3H, CH_3_), 2.34 (bs, 4H, 2CH_2_), 2.45 (bs, 4H, 2CH_2_), 2.71 (m, 2H, CH_2_), 2.89 (m, 2H, CH_2_), 3.02 (m, 2H, CH_2_), 3.41 (s, 2H, CH_2_), 3.65 (d, *J* = 6.68 Hz, 1H, CH), 3.80 (m, 2H, CH_2_), 6.66 (d, *J* = 8.83 Hz, 2H, ArH, H-2′ and H-6′), 6.80 (d, *J* = 8.83 Hz, 2H, ArH, H-3′ and H-5′), 7.28 (m, 1H, ArH, H-5″), 7.33 (1H, ArH, H-6″), 7.46 (1H, ArH, H-2″), 8.90 (s, 1H, OH). ^13^C-NMR (126 MHz, DMSO-*d*_6_): (δ) 9.6 (CH_3_), 41.5 (C-2a and C-6a), 45.3 (C-3a and C-5a), 50.4 (C-3b), 51.1 (C-5b), 52.9 (C-2b and C-6b), 58.9 (CH), 60.5 (CH_2_), 115.5 (C-2′ and C-6′), 116.6 (C-5″), 118.4 (C-3′ and C-5′), 119.1 (C-3″), 129.3 (C-6″), 130.5 (C-2″), 136.4 (C-1″), 144.1 (C-1′), 151.4 (C-4′), 156.2 (d, *J_C-F_* = 244.7 Hz, C-4″), 169.8 (C=O). Anal. Calculated for C_24_H_30_ClFN_4_O_2_: C 62.53, H 6.56, N 12.15. Found: C 62.57, H 6.53, N 12.10.

#### 3.3.4. General Procedure for the Synthesis of the 4*H*-1,2,4-Triazol-4-amines (**3b–d**)

To a mixture of suitable 4-amino-4*H*-1,2,4-triazole-3-thiol (**10a** or **10b**, 1.03 mmol) and NaOH (41 mg, 1.03 mmol) in MeOH (8 mL), the suitable fluorobenzylbromide derivative **11a** or **11b** (1.03 mmol) was added dropwise. The pure compounds **3b–d** were obtained following the procedure already reported for parent compound **3a** [14].

3-(3-Chloro-4-fluorobenzylthio)-5-(pyridin-4-yl)-4*H*-1,2,4-triazol-4-amine (**3b**)

Yield: 100%. Yellow powder. M.p. 164–165 °C. ^1^H NMR (500 MHz, DMSO-*d*_6_): (δ) 4.46 (s, 2H, CH_2_), 6.28 (s, 2H, NH_2_), 7.36 (t, *J* = 9.1 Hz, 1H, ArH, H-5″), 7.48 (m, 1H, ArH, H-6″), 7.70 (d, *J* = 7.1 Hz, 1H, ArH, H-2″), 8.00 (d, *J* = 3.2 Hz, 2H, pyridine, H-2′ and H-6′), 8.72 (d, *J* = 3.2 Hz, 2H, pyridine, H-3′ and H-5′). ^13^C NMR (126 MHz, DMSO-*d*_6_): (δ) 33.4 (CH_2_), 116.8 (C-3″), 119.1 (C-5″), 121.3 (C-2′ and C-6′), 129.8 (C-2″), 131.1 (C-1″), 133.9 (C-6″), 135.9 (C-1′), 150.1 (C-3′ and C-5′), 152.1 (C-3), 154.3 (C-5), 156.4 (C-4″, d, *J_C-F_* = 246 Hz). Anal. Calculated for C_14_H_11_ClFN_5_S: C 50.08, H 3.30, N 20.86. Found: C 51.10, H 3.76, N 20.31.

3-((2-Chloro-4-fluorobenzyl)thio)-5-(pyridin-4-yl)-4H-1,2,4-triazol-4-amine (**3c**)

Yield 100%. Whitish powder. M.p. 171–172 °C. ^1^H NMR (500 MHz, DMSO-*d*_6_): (δ) 4.52 (s, 2H, CH_2_), 6.27 (s, 2H, NH_2_), 7.20 (m, 1H, ArH, H-5″), 7.49 (m, 1H, ArH, H-3″), 7.66 (m, 1H, ArH, H-6″), 8.00 (dd, *J* = 4.5, 1.6 Hz, 2H, pyridine, H-2′ and H-6′), 8.73 (dd, *J* = 4.5, 1.6 Hz, 2H, pyridine, H-3′ and H-5′). ^13^C-NMR (126 MHz, DMSO-d6): (δ) 32.3 (CH_2_), 114.4 (C-5″), 116.8 (C-3″), 121.4 (C-2′ and C-6′), 131.3 (C-1″), 132.9 (C-6″), 133.9 (C-1′), 134.1 (C-2″), 150.1 (C-3′ and C-5′), 152.2 (C-3), 153.9 (C-5), 161.4 (C-4″, d, *J_C-F_* = 246.4 Hz). C_14_H_11_ClFN_5_S Anal. Calculated for C_14_H_11_ClFN_5_S: C 50.08, H 3.30, N 20.86. Found: C 50.38, H 3.60, N 21.16.

3-(3-Chloro-4-fluorobenzylthio)-5-phenyl-4H-1,2,4-triazol-4-amine (**3d**)

Yield: 100%. White powder. M.p. 164–166 °C. ^1^H NMR (500 MHz, DMSO-*d*_6_): (δ) 4.43 (s, 2H, CH_2_), 6.17 (bs, 2H, NH_2_), 7.36 (t, *J* = 9.0 Hz, 1H, ArH, H-5″), 7.47 (m, 1H, ArH, H-6″), 7.50 (d, *J* = 5.14 Hz, 1H, ArH, H-3″ and 5″), 7.69 (d, *J* = 7.15 Hz, 1H, ArH, H-2′), 7.97 (m, 2H, ArH, H-2′ and H-6′).^13^C NMR (126 MHz, DMSO-*d*_6_): (δ) 33.5 (CH_2_); 116.8 (C-5″); 119.1 (C-3″); 126.2 (C-2′ and C-6′); 127.9 (C-1′); 128.5 (C-3′ and C-5′); 130.5 (C-6″); 130.9 (C-4′); 131.2 (C-1″); 135.9 (C-2″); 153.1 (C-3); 154.1 (C-5); 156.4 (C-4″, d, *J_C-F_* = 246 Hz). Anal. Calculated for C_15_H_12_ClFN_4_S: C 53.81, H 3.61, N 16.73. Found: C 53.87, H 3.68, N 16.75.

#### 3.3.5. General Procedures for the Synthesis of Dithioacetals **4b** and **4f**

To a stirred solution of thiophenol (1.2 mmol) in dry isopropanol, NaOH (2.4 mmol) was added. After stirring the reaction at 80 °C under argon for 1 h, it was cooled to rt and diiodomethane was added (1.2 mmol). The mixture was then stirred for a further hour at 80 °C under argon. Afterwards, it was cooled to rt, the solvent was removed under reduced pressure, and the mixture was quenched with water (5 mL). The extraction of the water phase was carried out with EtAOc (3 × 5 mL), while the organic phase was washed with water (2 × 5 mL) and brine (2 × 5 mL) and dried over Na_2_SO_4_. The solvent was concentrated in vacuo and the crude compounds were purified through column chromatography on silica gel (96:4 *v*/*v*, petroleum ether/dichloromethane).

Phenylsulfanylmethylsulfanylbenzene (**4b**)

Yield: 93%. White solid. M.p. 34–35 °C. ^1^H NMR (400 MHz, CDCl_3_): (δ) 4.35 (s, 2H, SCH_2_), 7.25 (m, 2H, ArH, H-4′), 7.32 (m, 4H, ArH, H-3′ and H-5′), 7.43 (m, 4H, ArH, H-2′ and H-6′). ^13^C NMR (100 MHz, CDCl_3_): (δ) 40.6 (SCH_2_), 127.1 (C-4), 129.0 (C-3 and C-5), 130.7 (C-2 and C-6), 135.0 (C-1). Anal. Calculated for C_13_H_12_S_2_: C 67.20, H 5.21. Found: C 67.24, H 5.19.

2-Chloro-4-[(3-chloro-4-fluorophenyl)sulfanylmethylsulfanyl]-1-fluorobenzene (**4f**)

Yield: 93%. Colorless oil. ^1^H NMR (600 MHz, CDCl_3_): (δ) 4.24 (s, 2H, SCH_2_), 7.09 (m, 2H, ArH), 7.29 (m, 2H, ArH), 7.46 (m, 2H, ArH). ^13^C NMR (150 MHz, CDCl_3_): (δ) 42.5 (SCH_2_), 117.1 (d, *J* = 21.6 Hz), 121.6 (d, *J* = 18.6 Hz), 130.6 (d, *J* = 3.5 Hz), 131.8 (d, *J* = 7.2 Hz), 133.9, 157.8 (d, *J_C-F_* = 249.5 Hz). Anal. Calculated for C_13_H_8_C_l2_F_2_S_2_: C 46.30, H 2.39. Found: C 46.27, H 2.20.

#### 3.3.6. Synthesis of 1-[(Chloromethyl)sulfanyl]-4-fluorobenzene (**14c**)

To a mixture of toluene (5 mL) and HCl (37%, 5 mL), paraformaldehyde was added (152 mg, 5.1 mmol). The resulting mixture was stirred at 50 °C for 10 min and then a solution of 4-fluorobenzene-1-thiol (13) (500 mg, 3.9 mmol) in toluene (5 mL) was added dropwise at the same temperature. The reaction was stirred at 50 °C for 1 h and then at room temperature for 3 h and then flushed with Ar, and the layers were separated. The water phase was extracted with DCM (3 × 10 mL) and the organic phase was concentrated in vacuo to obtain the desired compound, which was used without further purification.

Yield: 90%. Yellow oil. ^1^H NMR (600 MHz, CDCl_3_): (δ) 4.89 (s, 2H, SCH_2_Cl), 7.08 (m, 2H, Ar H), 7.54 (m, 2H, ArH). ^13^C NMR (150 MHz, CDCl_3_): (δ) 52.0 (SCH_2_Cl), 128.1 (d, *J* = 2.8 Hz), 116.4 (d, *J* = 21.9 Hz), 134.3 (d, *J* = 8.4 Hz), 163.0 (d, *J_C-F_* = 249.6 Hz). Anal. Calculated for C_7_H_6_ClFS: C 47.60, H 3.42. Found: C 47.66, H 3.47.

#### 3.3.7. General Procedure for the Preparation of the Dithioacetals **4c–e** and **4g–h**

To a stirred solution of the suitable chloro(methylsulfanyl) derivative (1.0 mmol) (**14a–c**) and K_2_CO_3_ (2.0 mmol) in DMF (5 mL), the proper thiophenol (1.5 mmol) was added. The reaction was stirred at room temperature overnight and then quenched with water and extracted with Et_2_O (3 × 5 mL). The organic phase was washed with brine and concentrated in vacuo. The desired products **4c–e** and **4g–h** were obtained after purification through column chromatography on silica gel (96:4 *v*/*v*, petroleum ether/dichloromethane).

1-Fluoro-4-{[(phenylsulfanyl)methyl]sulfanyl}benzene (**4c**)

Yield: 91%. Yellow oil. ^1^H NMR (400 MHz, CDCl_3_): (δ) 4.29 (s, 2H, SCH_2_), 7.01 (m, 2H, ArH, H-2′ and H-6′), 7.26 (m, 1H, ArH, H-4″), 7.32 (m, 2H, ArH, H-3′ and H-5″), 7.41 (m, 2H, ArH, H-2″ and H-6″), 7.44 (m, 2H, ArH, H-3′ and H-5″). ^13^C NMR (100 MHz, CDCl_3_): (*δ*) 41.9 (d, *J* = 1.2 Hz, SCH_2_), 116.1 (d, *J* = 21.9 Hz, C’2 and C-6′), 127.1 (C″-4), 129.0 (C’’-3 and C’’-5), 129.6 (d, *J* = 3.3 Hz, C’-4), 130.7 (C-2″ and C-6″), 134.1 (d, *J* = 8.2 Hz, C-3′), 134.2 (d, *J* = 8.2 Hz, C-5′), 134.8 (C-1″), 162.5 (d, *J_C-F_* = 247.9 Hz). Anal. Calculated for C_13_H_11_FS_2_: C 62.37, H 4.43. Found: C 62.53, H 4.48.

2-Chloro-1-fluoro-4-{[(phenylsulfanyl)methyl]sulfanyl}benzene (**4d**)

Yield: 87%. Colorless oil. ^1^H NMR (600 MHz, CDCl_3_): (δ) 4.29 (s, 2H, SCH_2_), 7.28 (m, 1H, ArH), 7.32 (m, 2H, ArH), 7.41 (m, 2H, ArH), 7.47 (m, 1H, ArH). ^13^C NMR (150 MHz, CDCl_3_): (*δ*) 41.7 (SCH_2_), 117.0 (d, *J* = 21.7 Hz), 121.4 (d, *J* = 17.6 Hz), 127.4, 129.1, 131.0, 131.1 (d, *J* = 4.1 Hz), 131.7 (d, *J* = 7.1 Hz), 133.7, 134.3, 157.7 (d, *J_C-F_* = 248.4 Hz). C_13_H_10_ClFS_2_ Anal. Calculated for C_13_H_10_ClFS_2_: C 54.83, H 3.54. Found: C 54.90, H 3.55.

2-Chloro-1-fluoro-4-(([(4-fluorophenyl)sulfanyl]methyl)sulfanyl)benzene (**4e**)

Yield: 94%. Colorless oil. ^1^H NMR (600 MHz, CDCl_3_): (δ) 4.23 (s, 2H, SCH_2_), 7.02 (m, 2H, Ph H), 7.08 (m, 1H, ArH), 7.28 (m, 1H, ArH), 7.43 (m, 3H, ArH). ^13^C NMR (150 MHz, CDCl_3_): (δ) 42.8 (SCH_2_), 116.2 (d, *J* = 21.2 Hz), 117.0 (d, *J* = 23.3 Hz), 121.4 (d, *J* = 19.6 Hz), 129.1 (d, *J* = 2.7 Hz), 131.0 (d, *J* = 4.3 Hz), 131.5 (d, *J* = 7.0 Hz), 133.6, 134.2 (d, *J* = 8.5 Hz), 157.6 (d, *J_C-F_* = 251.9 Hz), 162.6 (d, *J_C-F_* = 245.9 Hz). Anal. Calculated for C_13_H_9_ClF_2_S_2_: C 51.57, H 3.00. Found: 51.33, H 2.95.

1-Fluoro-4-{[(methylsulfanyl)methyl]sulfanyl}benzene (**4g**)

Yield: 88%. Colorless oil. ^1^H NMR (600 MHz, CDCl_3_): (δ) 2.22 (s, 3H, SCH_3_), 3.93 (s, 2H, SCH_2_), 7.02 (m, 2H, ArH), 7.44 (m, 2H, ArH). ^13^C NMR (150 MHz, CDCl_3_): (δ) 15.0 (SCH_3_), 41.8 (SCH_2_), 116.0 (d, *J* = 21.9 Hz), 129.8 (d, *J* = 2.8 Hz), 133.9 (d, *J* = 8.0 Hz), 162.4 (d, *J_C-F_* = 245.7 Hz). Anal. Calculated for C_8_H_9_FS_2_: C 51.04, H 4.82. Found: C 51.34, H 4.75.

1-Fluoro-3-{[(methylsulfanyl)methyl]sulfanyl}benzene (**4h**)

Yield: 94%. Brown oil. ^1^H NMR (400 MHz, CDCl_3_): (δ) 2.24 (s, 3H, SCH_3_), 4.01 (s, 2H, SCH_2_S), 6.92 (m, 1H, ArH, H-6), 7.13 (m, 1H, ArH, H-2), 7.17 (m, 1H, ArH, H-4), 7.27 (m, 1H, ArH, H-5). ^13^C NMR (100 MHz, CDCl_3_): (δ) 15.2 (SCH_3_), 39.8 (SCH_2_S), 113.7 (d, *J* = 21.2 Hz, C-6), 116.7 (d, *J* = 23.0 Hz, C-2), 125.4 (d, *J* = 3.0 Hz, C-4), 130.1 (d, *J* = 8.5 Hz, C-5), 137.6 (d, *J* = 7.8 Hz, C-3), 162.7 (d, *J_C-F_* = 248.4 Hz). Anal. Calculated for C_8_H_9_FS_2_: C 51.04, H 4.82. Found: C 51.11, H 4.78.

## 4. Conclusions

In summary, potent inhibitors of TYR from *Agaricus bisporus* were identified through very simple modifications of 4-fluorophenyl-based compounds that have been previously described. This investigation confirmed that the 3-chloro-4-fluorophenyl moiety was a fragment able to enhance the AbTYR inhibitory activity of distinct chemotypes of molecules targeting AbTYR. Our docking studies suggested that the improvement in activity might be due to additional interactions within the enzyme cavity that are able to reinforce the fluorine bond with crucial residues.

## Data Availability

Not applicable.

## Supplementary Materials

The following supporting information can be downloaded at https://www.mdpi.com/article/10.3390/ijms24097944/s1.
