# Supplementary material for: Leveraging the 3-Chloro-4-fluorophenyl Motif to Identify Inhibitors of Tyrosinase from Agaricus bisporus
Source: Int J Mol Sci. 2023 Apr 27;24(9):7944. doi: 10.3390/ijms24097944 (PMC10177926; doi:10.3390/ijms24097944)
Supplement: Supplementary file 1 [file ijms-24-07944-s001.zip › ijms-2356159-supplementary.pdf]

Leveraging the 3-Chloro-4-fluorophenyl Motif to Identify Inhibitors of Tyrosinase from *Agaricus bisporus*

Salvatore Mirabile [1,2], Laura Ielo [3], Lisa Lombardo [1], Federico Ricci [1], Rosaria Gitto [1], Maria Paola Germanò [1], Vittorio Pace [3,*] and Laura De Luca [1,*]

[1] Department of Chemical, Biological, Pharmaceutical and Environmental Sciences, University of Messina, Viale F. Stagno D'Alcontres 31, I-98166 Messina, Italy; salvatore.mirabile@unime.it (S.M.); lisa.lombardo@studenti.unime.it (L.L.); federico.ricci@unime.it (F.R.); rosaria.gitto@unime.it (R.G.); mariapaola.germano@unime.it (M.P.G.)

[2] Foundation Prof. Antonio Imbesi, University of Messina, Piazza Pugliatti 1, I-98122 Messina, Italy

[3] Department of Chemistry, University of Turin, Via P. Giuria 7, 10125 Torino, Italy; laura.ielo@unito.it

* Correspondence: vittorio.pace@unito.it (V.P.); laura.deluca@unime.it (L.D.L.); Tel. +39-011-6707994 (V.P.); 39-090-6766410 (L.D.L.)

Content:

Figures S1-S38: ^{1}H-NMR and ^{13}C-NMR spectra of synthetized compounds…………………………...........S2

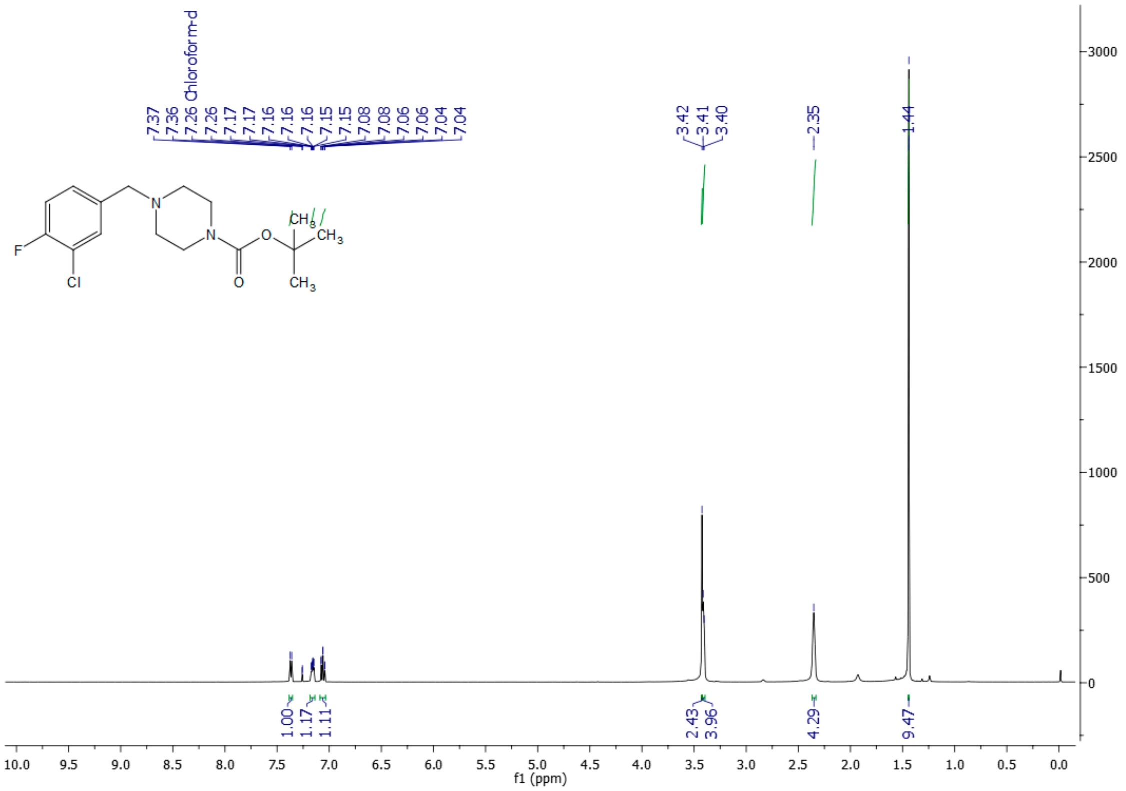

Figure S1: ^{1}H-NMR ($CDCl_3$) spectrum of *tert*-Butyl 4-[(3-chloro-4-fluorophenyl)methyl]piperazine-1-carboxylate

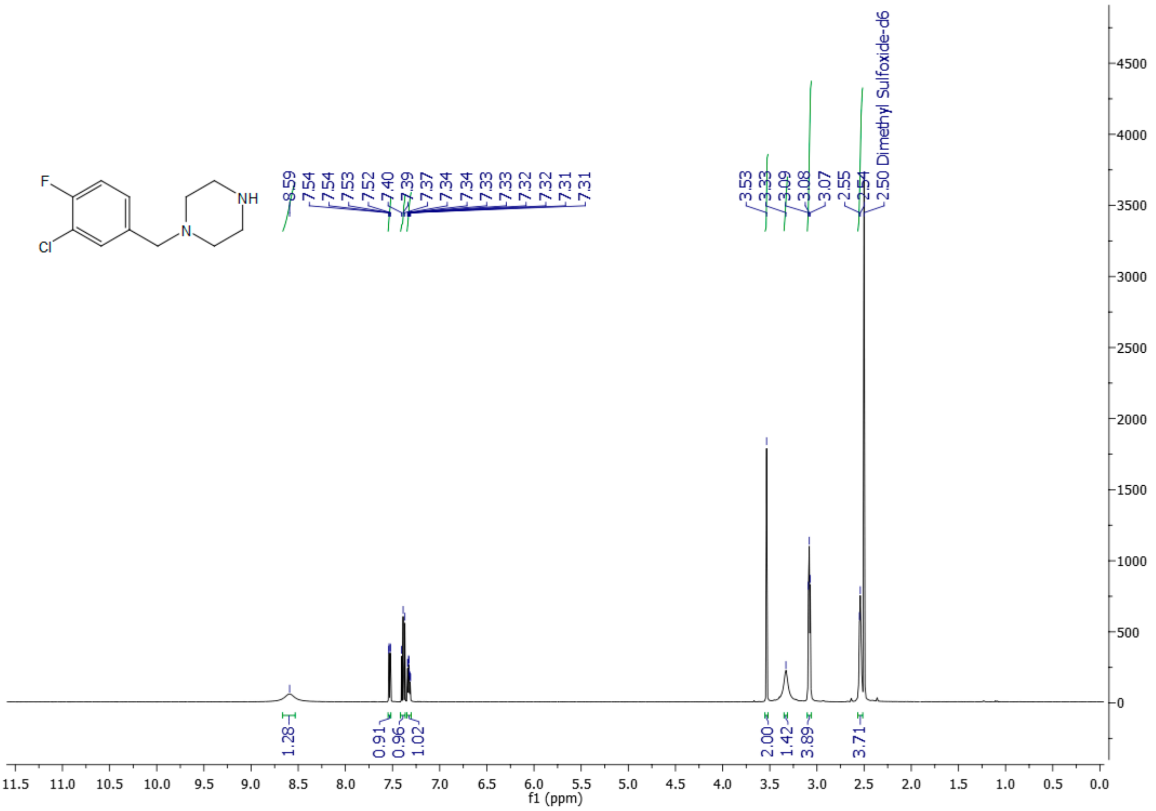

Figure S2: ^{1}H-NMR (DMSO-d$_6$) spectrum of 1-[(3-chloro-4-fluorophenyl)methyl]piperazine **(6)**

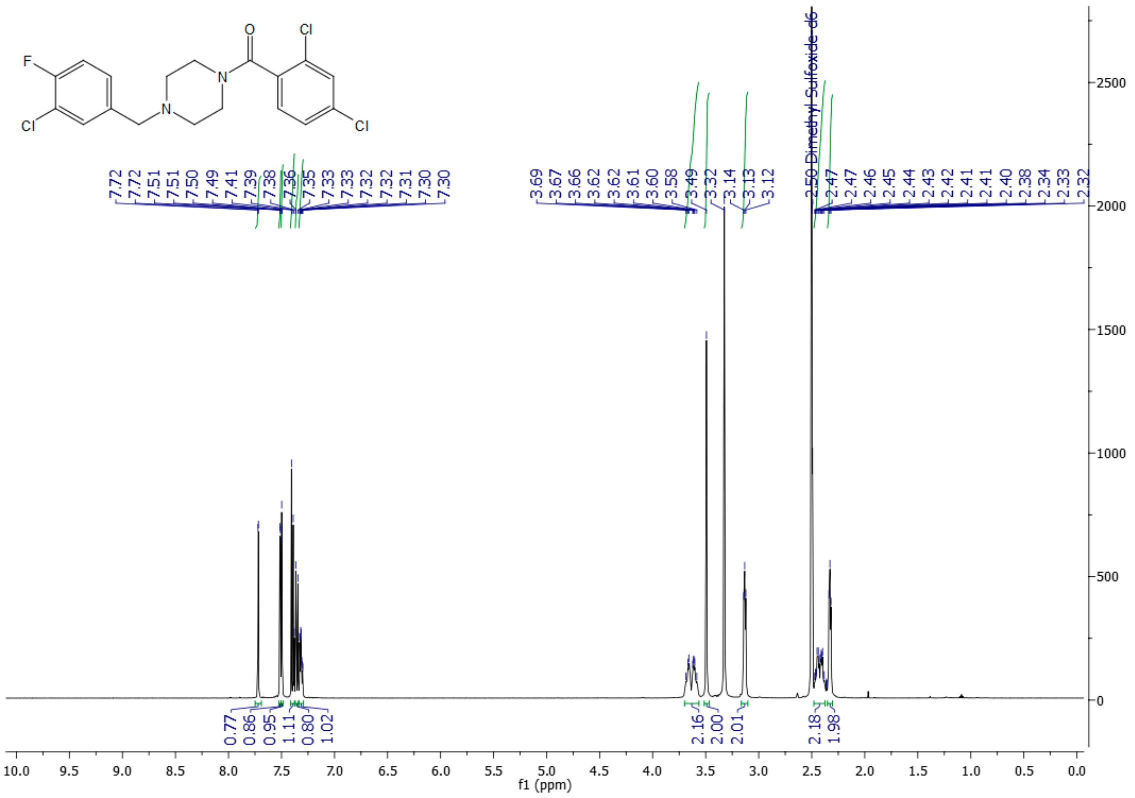

Figure S3: ^{1}H-NMR (DMSO-d$_6$) spectrum of (4-(3-Chloro-4-fluorobenzyl)piperazin-1-yl)(2,4-dichlorophenyl)methanone (**1d**)

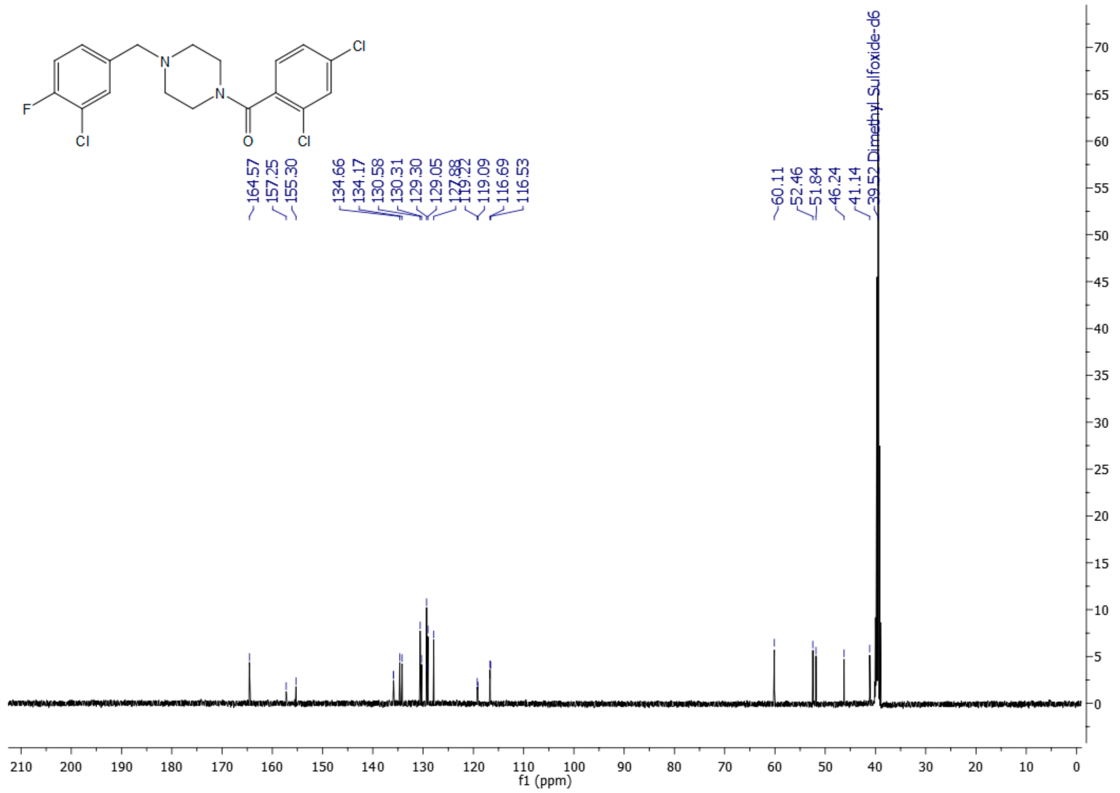

Figure S4: ^{13}C-NMR (DMSO-d_6) spectrum of (4-(3-Chloro-4-fluorobenzyl)piperazin-1-yl)(2,4-dichlorophenyl)methanone (**1d**)

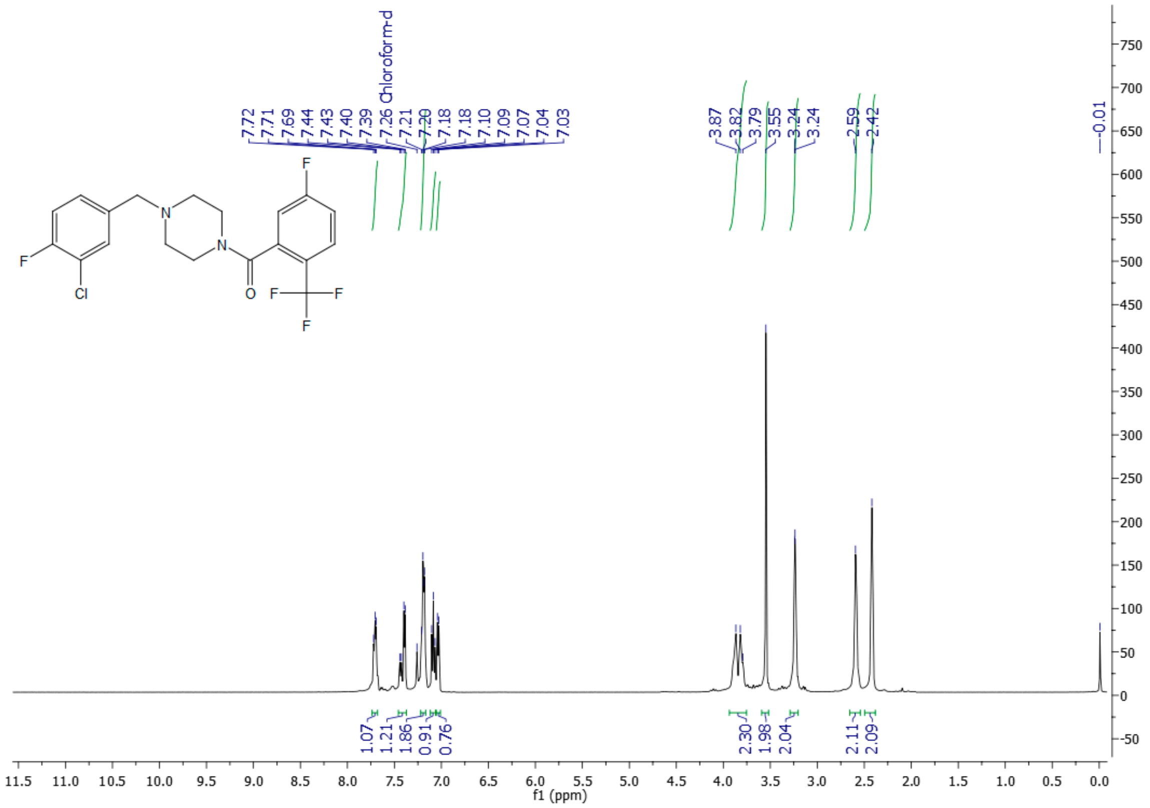

Figure S5: ^{1}H-NMR ($CDCl_3$) spectrum of (4-(3-Chloro-4-fluorobenzyl)piperazin-1-yl)(5-fluoro-2-(trifluoromethyl)phenyl)methanone (**1e**)

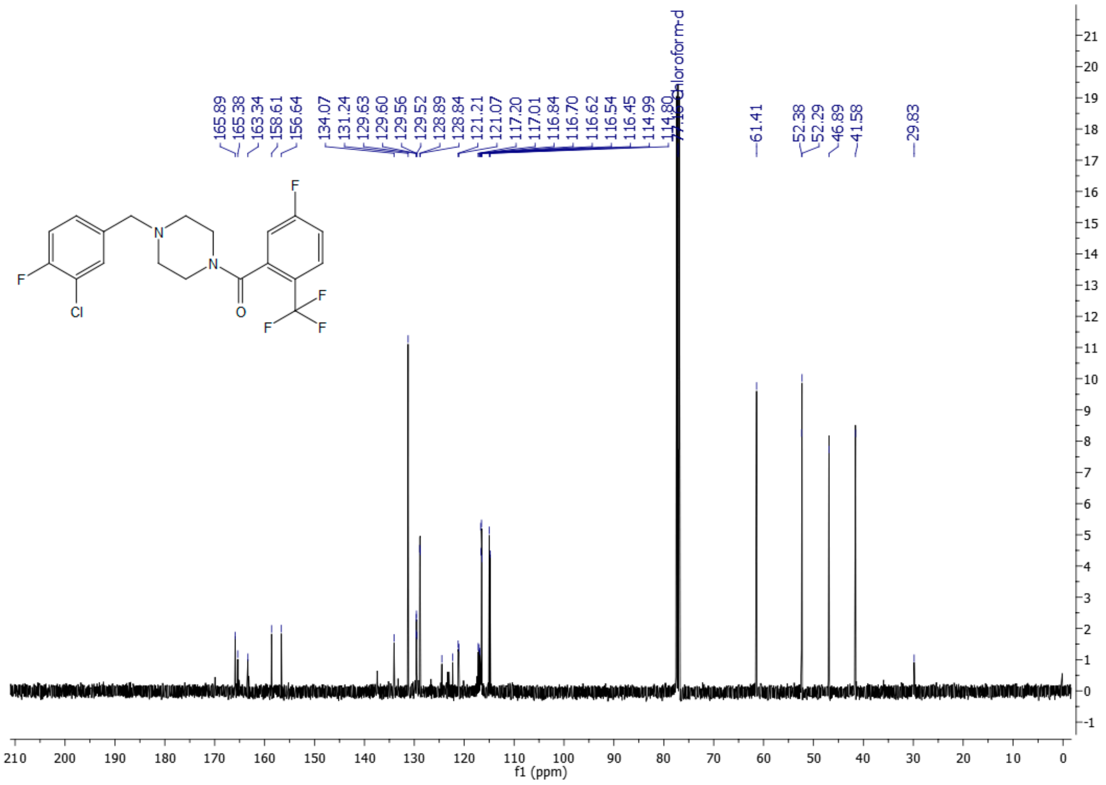

Figure S6: ^{13}C-NMR ($CDCl_3$) spectrum of (4-(3-Chloro-4-fluorobenzyl)piperazin-1-yl)(5-fluoro-2-(trifluoromethyl)phenyl)methanone (**1e**)

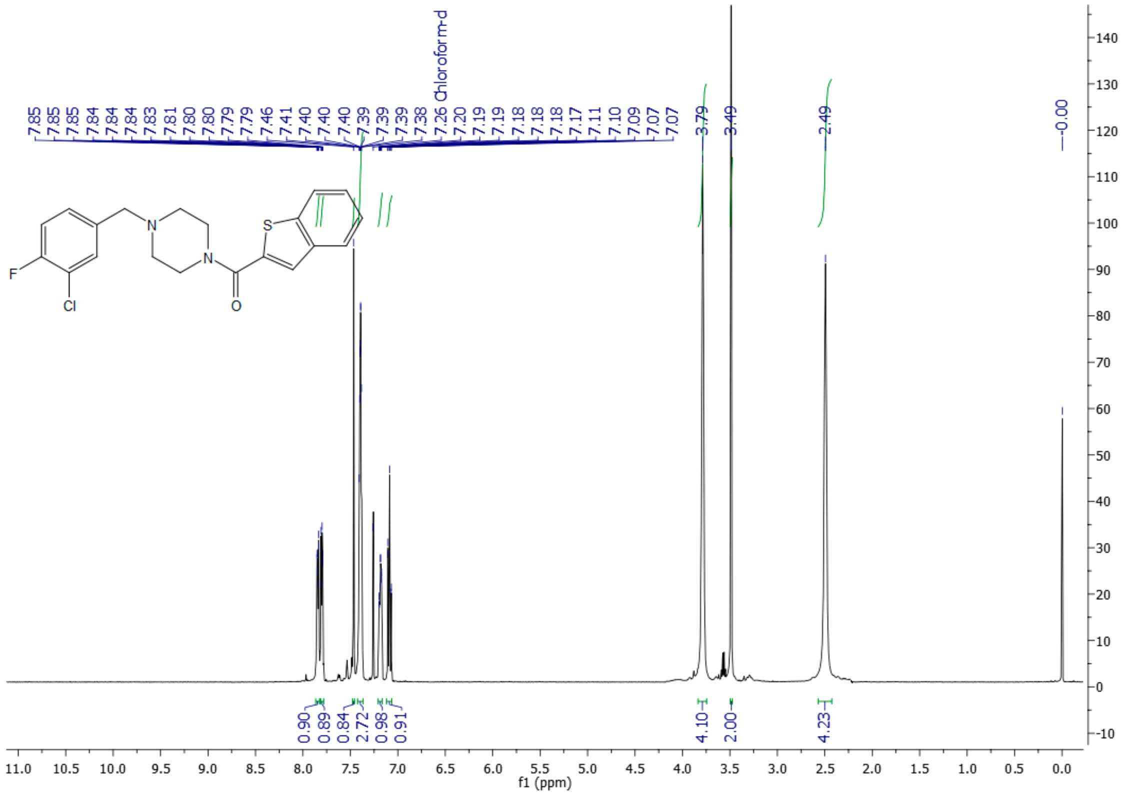

Figure S7: ^{1}H-NMR ($CDCl_3$) spectrum of Benzo[*b*]thiophen-2-yl(4-(3-chloro-4-fluorobenzyl)piperazin-1-yl)methanone (**1f**)

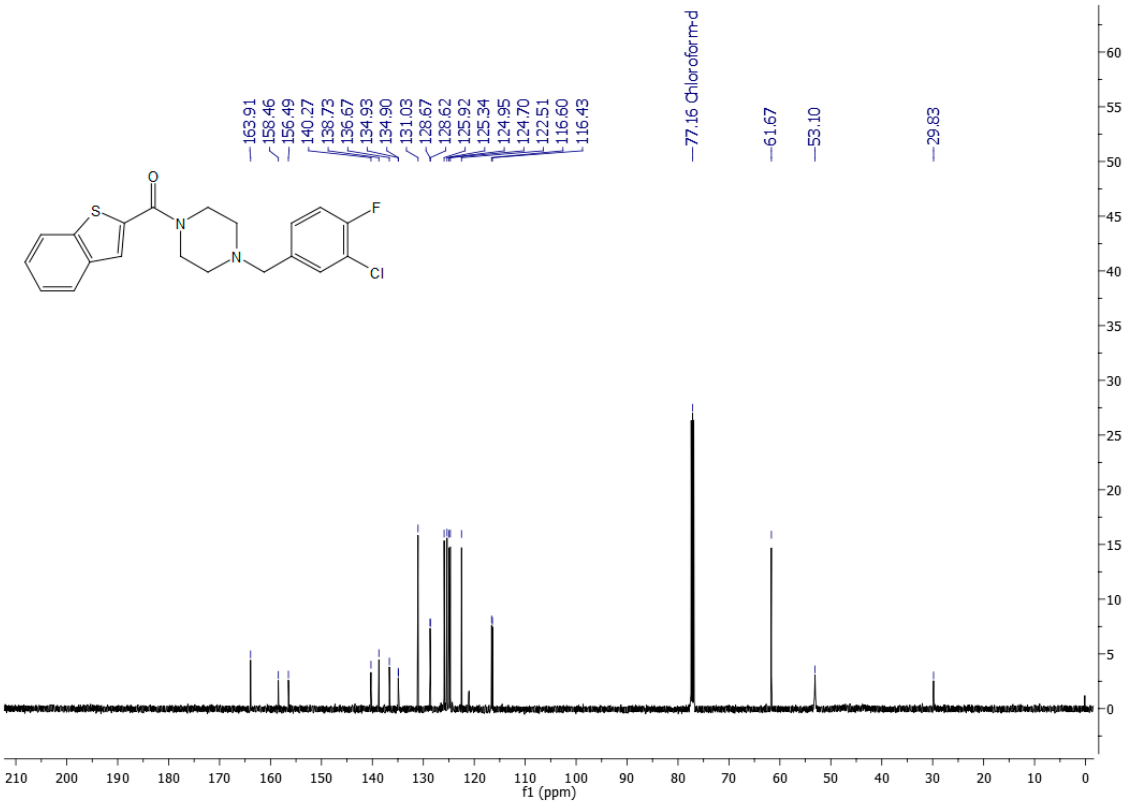

Figure S8: ^{13}C-NMR ($CDCl_3$) spectrum of Benzo[*b*]thiophen-2-yl(4-(3-chloro-4-fluorobenzyl)piperazin-1-yl)methanone (**1f**)

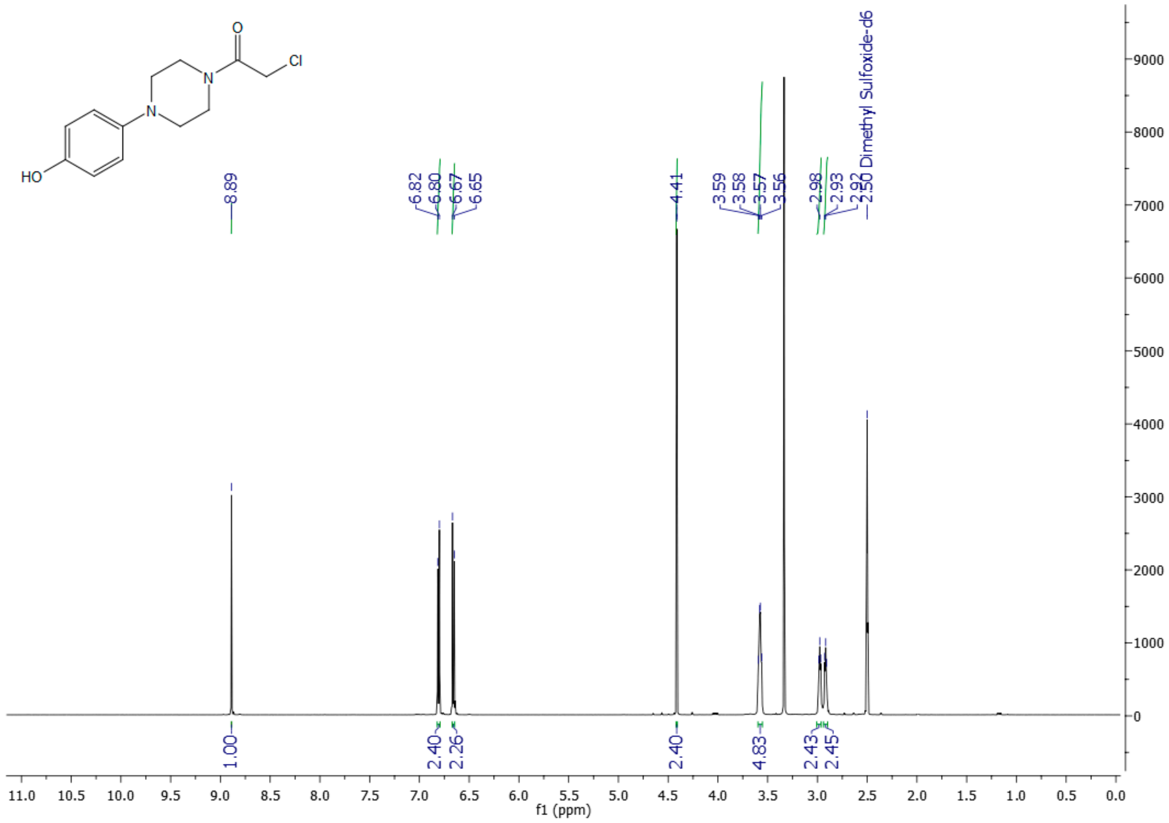

Figure S9: ^{1}H-NMR (DMSO-*d*6) spectrum of 2-Chloro-1-[4-(4-hydroxyphenyl)piperazin-1-yl]ethanone (**8a**)

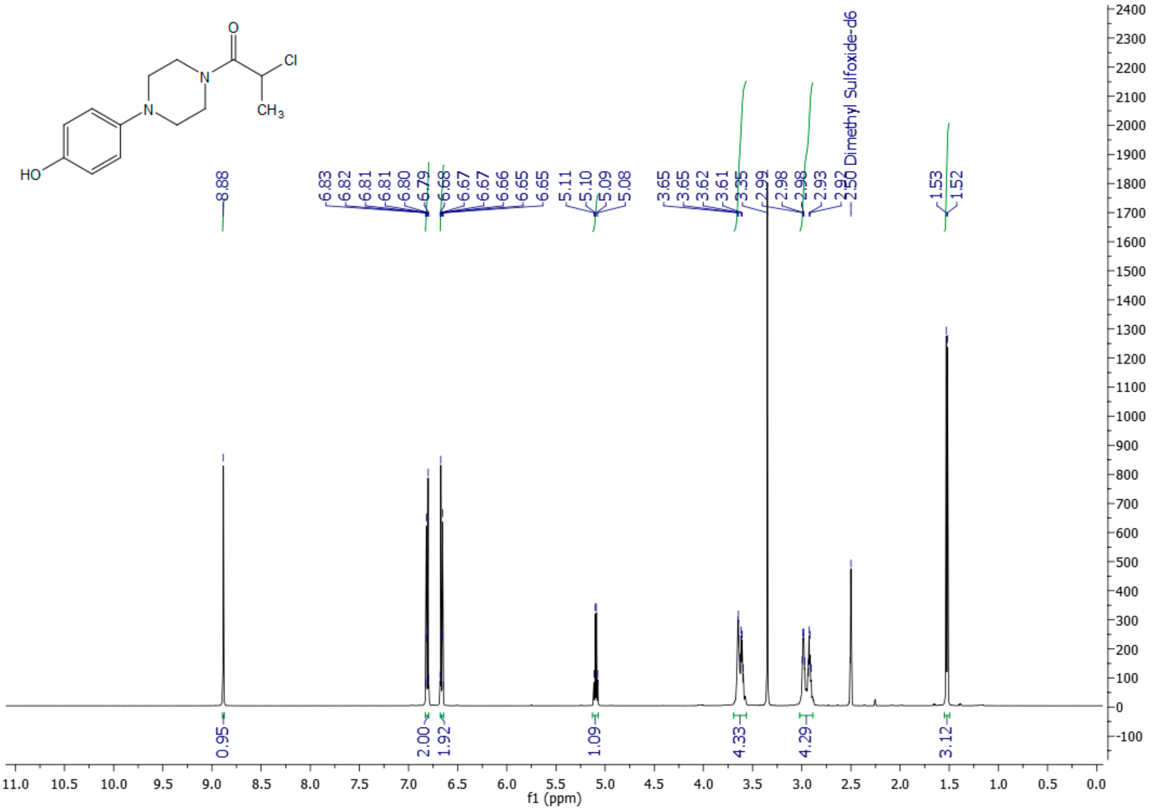

Figure S10: ^{1}H-NMR (DMSO-*d*6) spectrum of 2-Chloro-1-(4-(4-hydroxyphenyl)piperazin-1-yl)propan-1-one (**8b**)

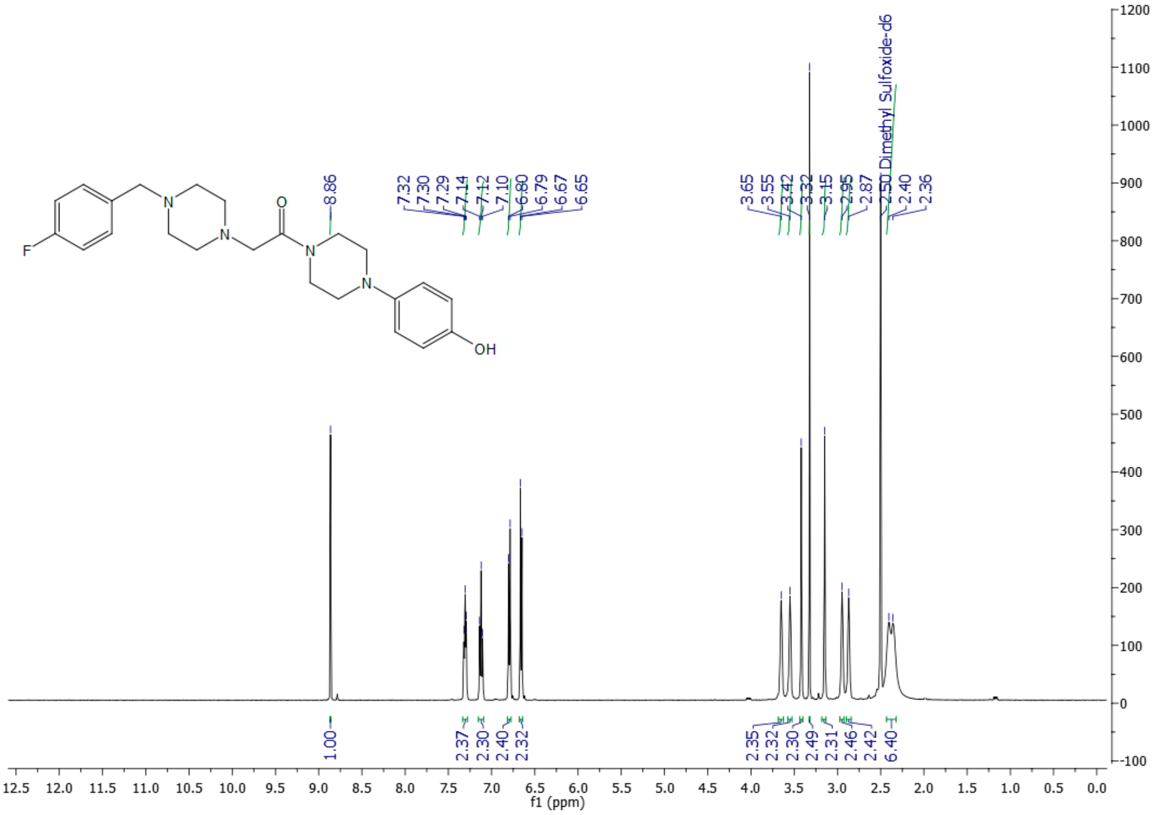

Figure S11: ^{1}H-NMR (DMSO-d_6) spectrum of 2-(4-(4-Fluorobenzyl)piperazin-1-yl)-1-(4-(4-hydroxyphenyl)piperazin-1-yl)ethanone (**2b**)

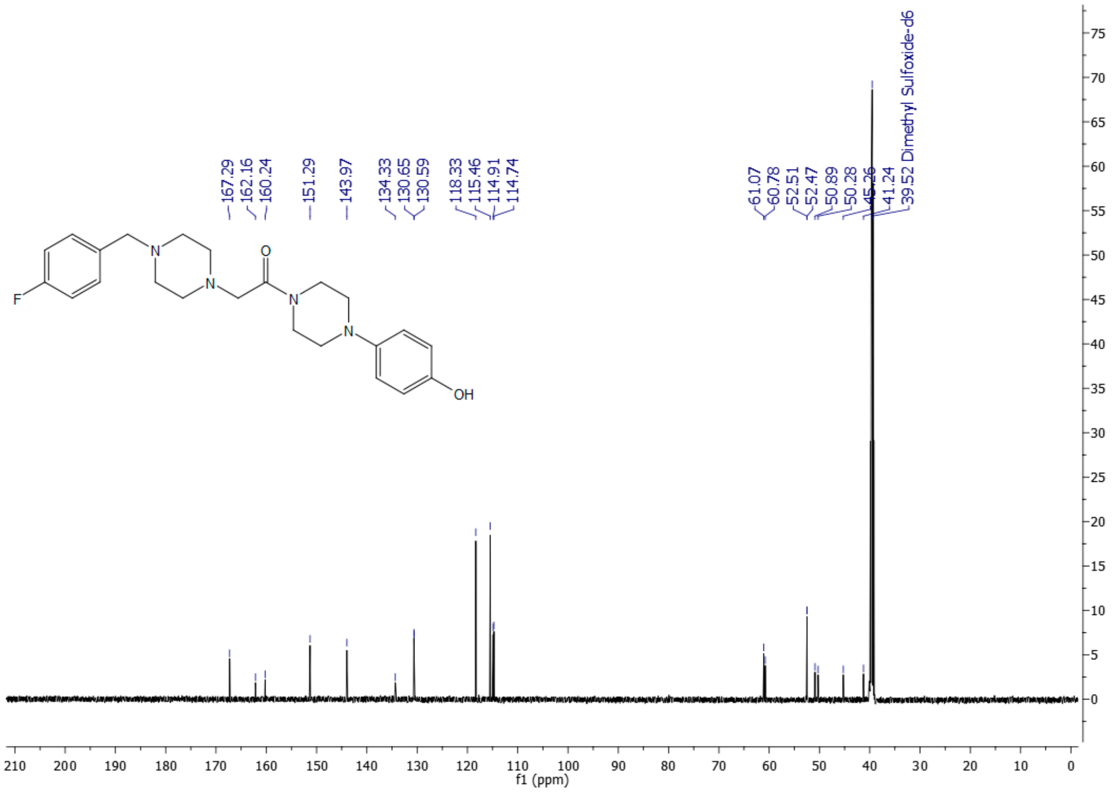

Figure S12: ^{13}C-NMR (DMSO-d_6) spectrum of 2-(4-(4-Fluorobenzyl)piperazin-1-yl)-1-(4-(4-hydroxyphenyl)piperazin-1-yl)ethanone (**2b**)

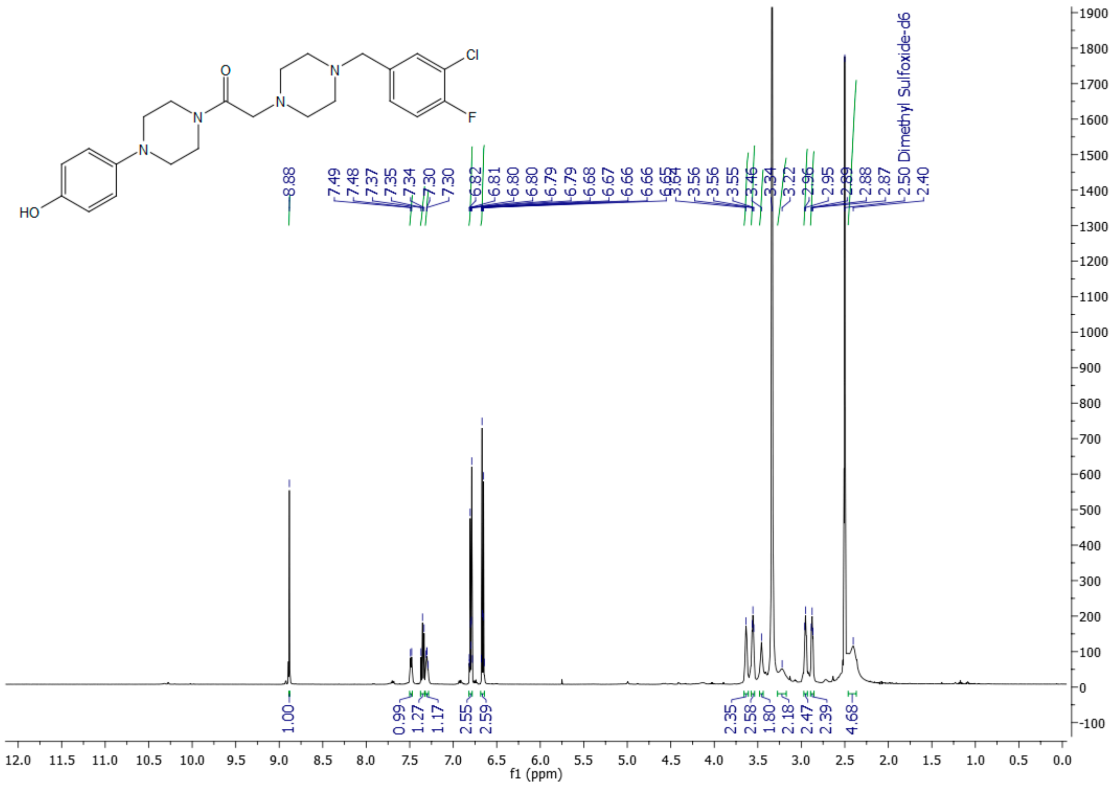

Figure S13: ^{1}H-NMR (DMSO-d_6) spectrum of 2-(4-(3-Chloro-4-fluorobenzyl)piperazin-1-yl)-1-(4-(4-hydroxyphenyl)piperazin-1-yl)ethanone (**2c**)

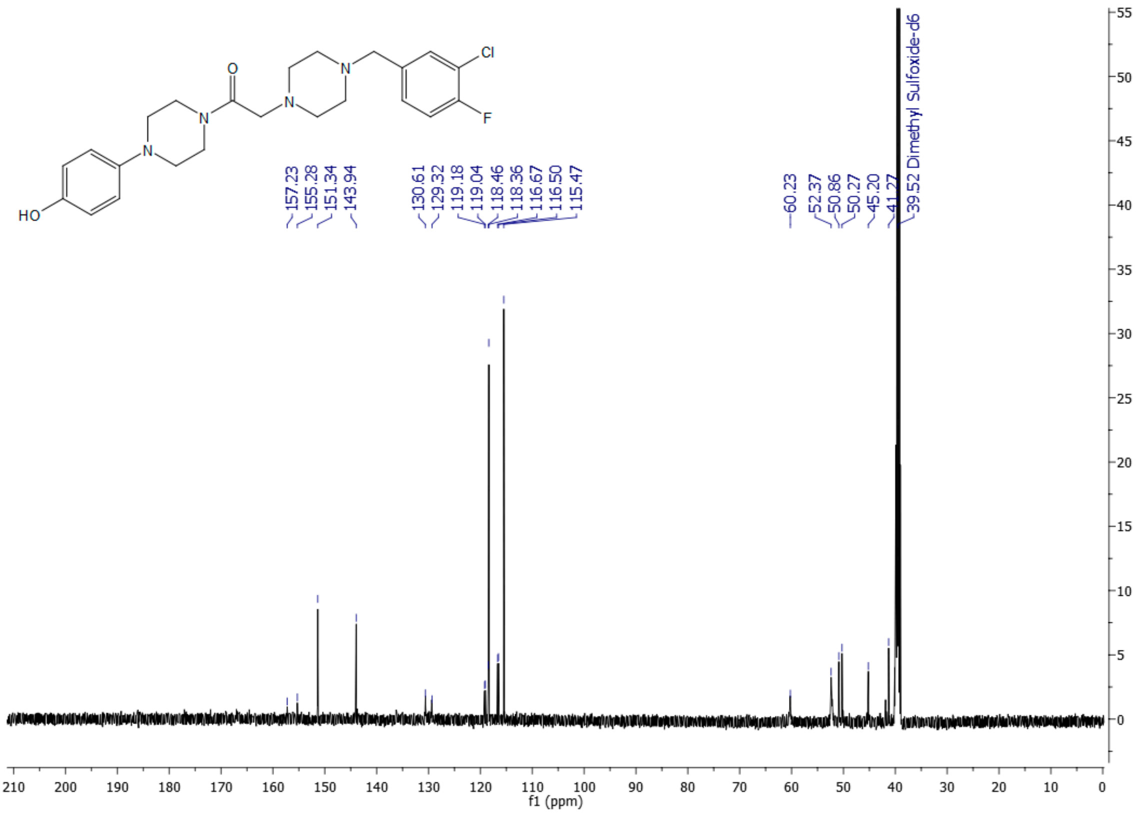

Figure S14: ^{13}C-NMR (DMSO-d_6) spectrum of 2-(4-(3-Chloro-4-fluorobenzyl)piperazin-1-yl)-1-(4-(4-hydroxyphenyl)piperazin-1-yl)ethanone (**2c**)

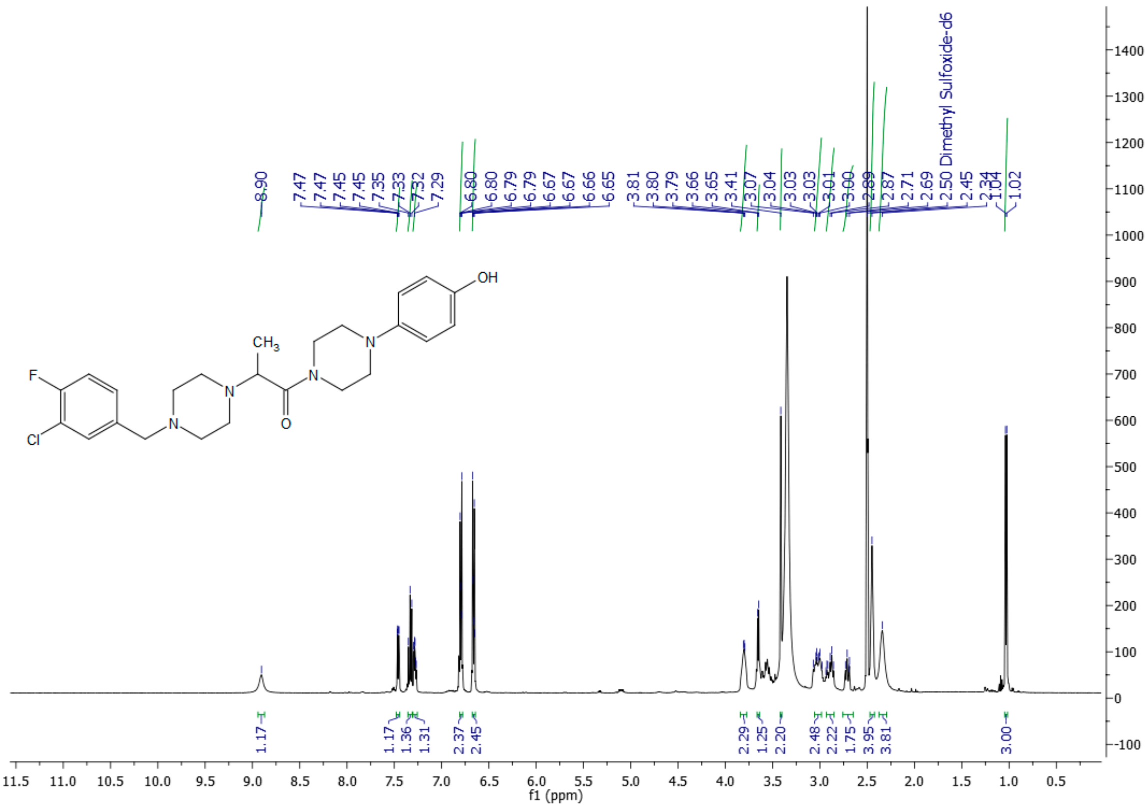

Figure S15: ^{1}H-NMR (DMSO-d_6) spectrum of 2-(4-(3-Chloro-4-fluorobenzyl)piperazin-1-yl)-1-(4-(4-hydroxyphenyl)piperazin-1-yl)propan-1-one (**2d**)

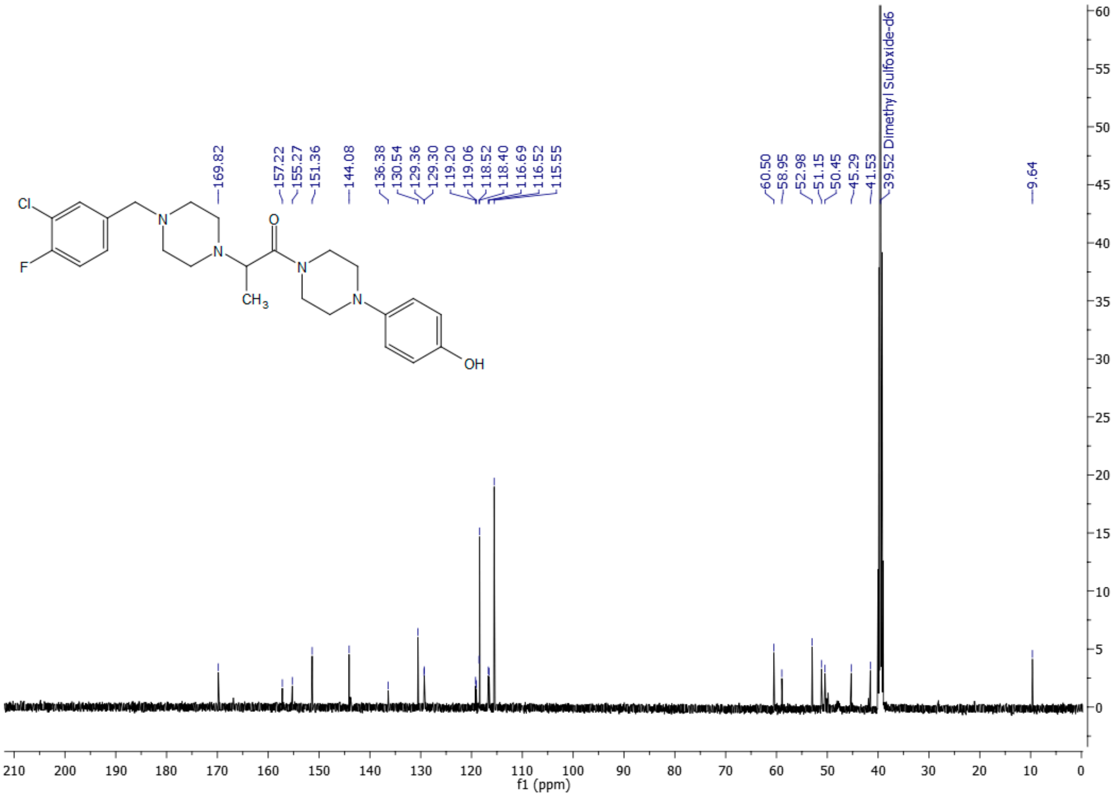

Figure S16: ^{13}C-NMR (DMSO-d_6) spectrum of 2-(4-(3-Chloro-4-fluorobenzyl)piperazin-1-yl)-1-(4-(4-hydroxyphenyl)piperazin-1-yl)propan-1-one (**2d**)

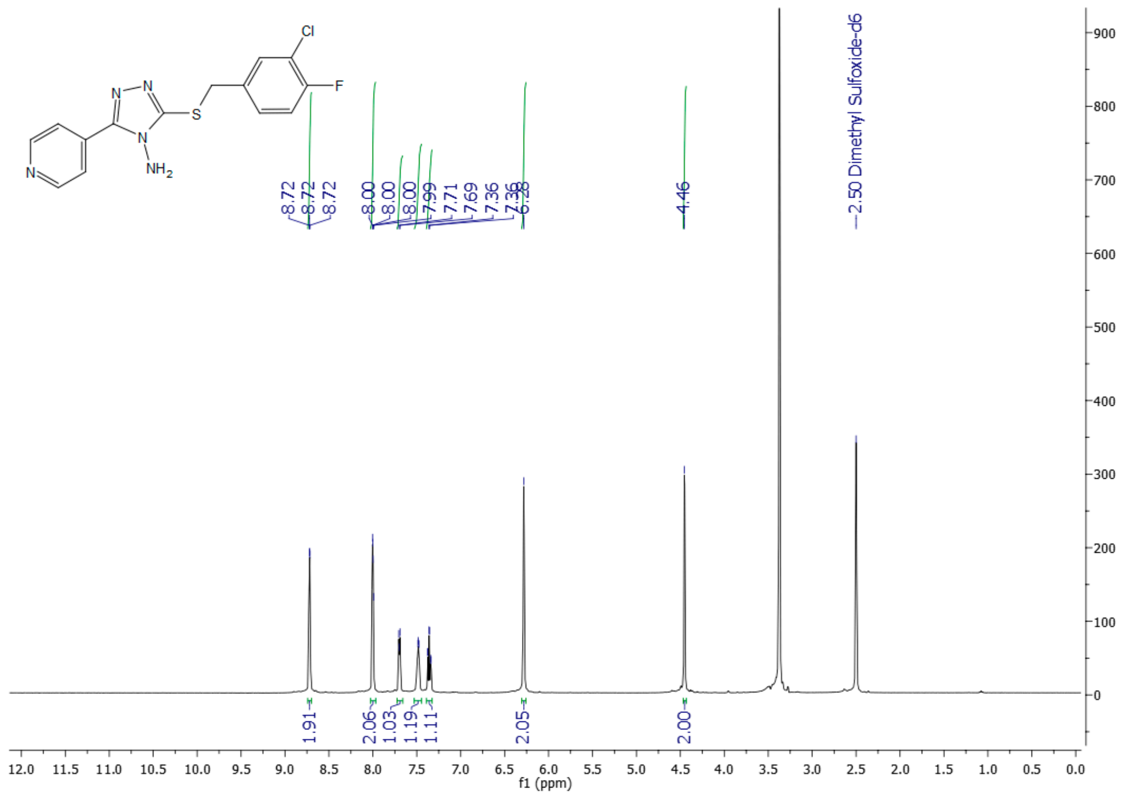

Figure S17: ^{1}H-NMR (DMSO-d_6) spectrum of 3-(3-Chloro-4-fluorobenzylthio)-5-(pyridin-4-yl)-4*H*-1,2,4-triazol-4-amine (**3b**)

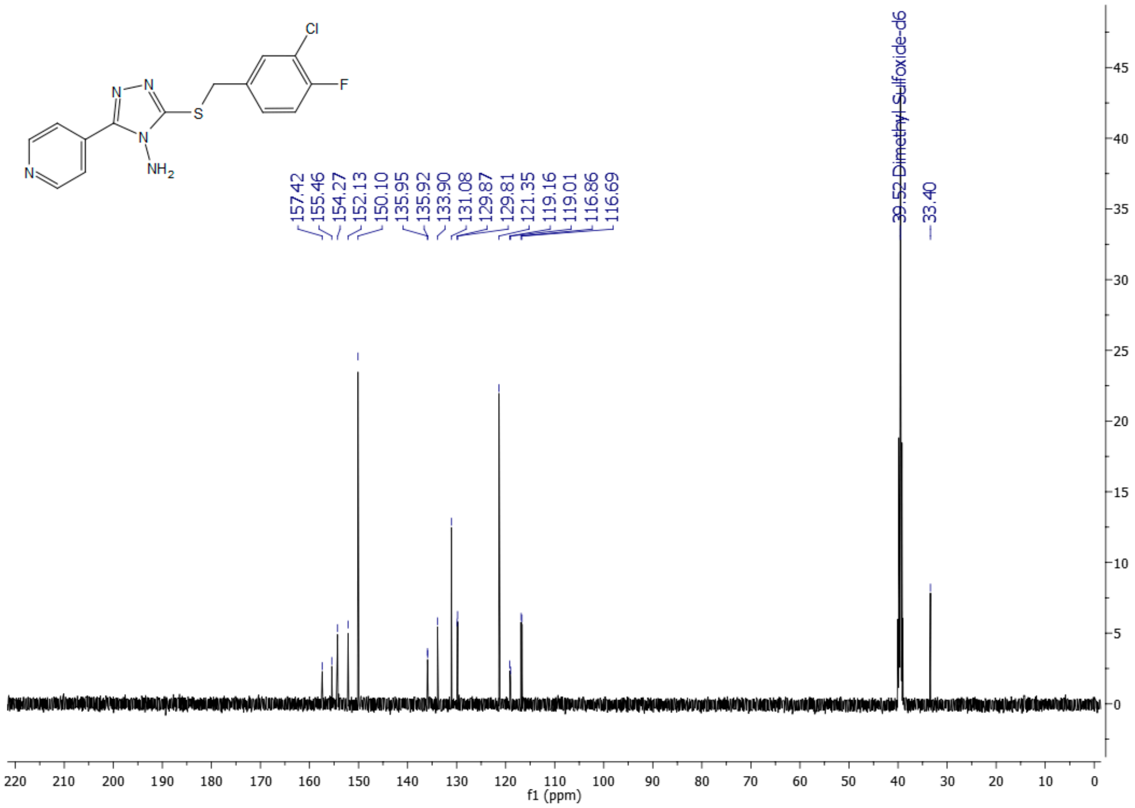

Figure S18: ^{13}C-NMR (DMSO-d_6) spectrum of 3-(3-Chloro-4-fluorobenzylthio)-5-(pyridin-4-yl)-4*H*-1,2,4-triazol-4-amine (**3b**)

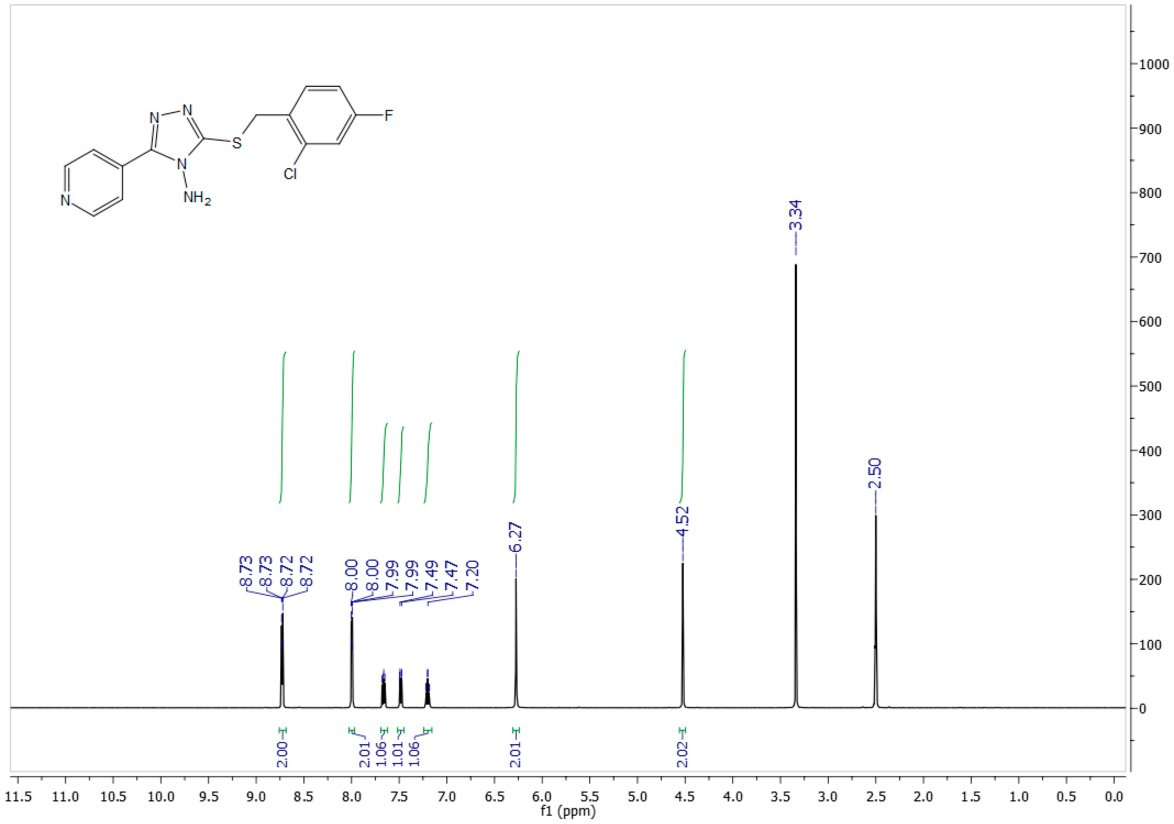

Figure S19: ^{1}H-NMR (DMSO-d_6) spectrum of 3-((2-Chloro-4-fluorobenzyl)thio)-5-(pyridin-4-yl)-4H-1,2,4-triazol-4-amine (**3c**)

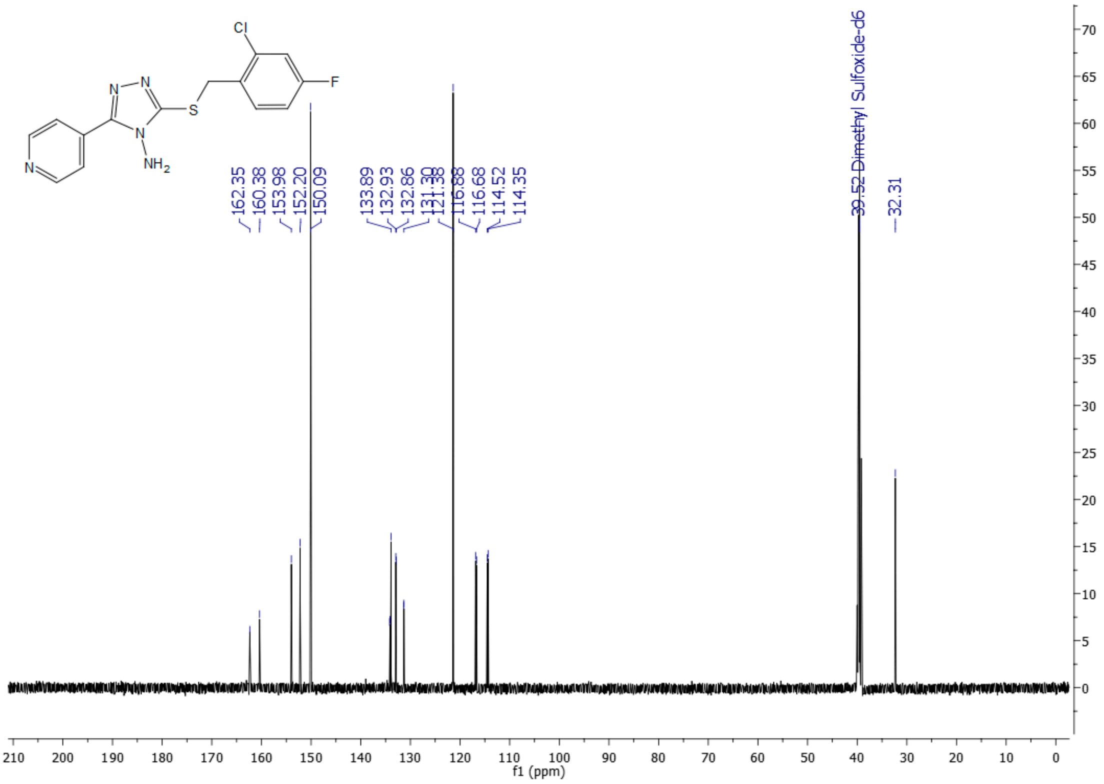

Figure S20: ^{13}C-NMR (DMSO-d_6) spectrum of 3-((2-Chloro-4-fluorobenzyl)thio)-5-(pyridin-4-yl)-4H-1,2,4-triazol-4-amine (**3c**)

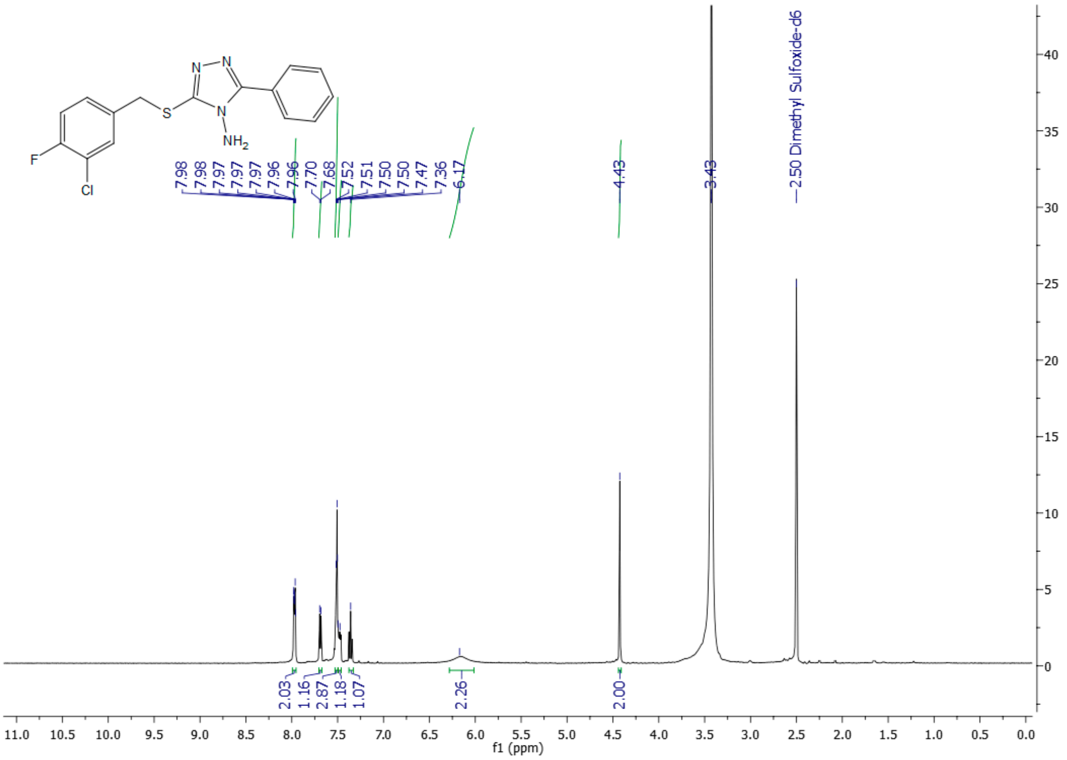

Figure S21: ^{1}H-NMR (DMSO-d_6) spectrum of 3-(3-Chloro-4-fluorobenzylthio)-5-phenyl-4H-1,2,4-triazol-4-amine (**3d**)

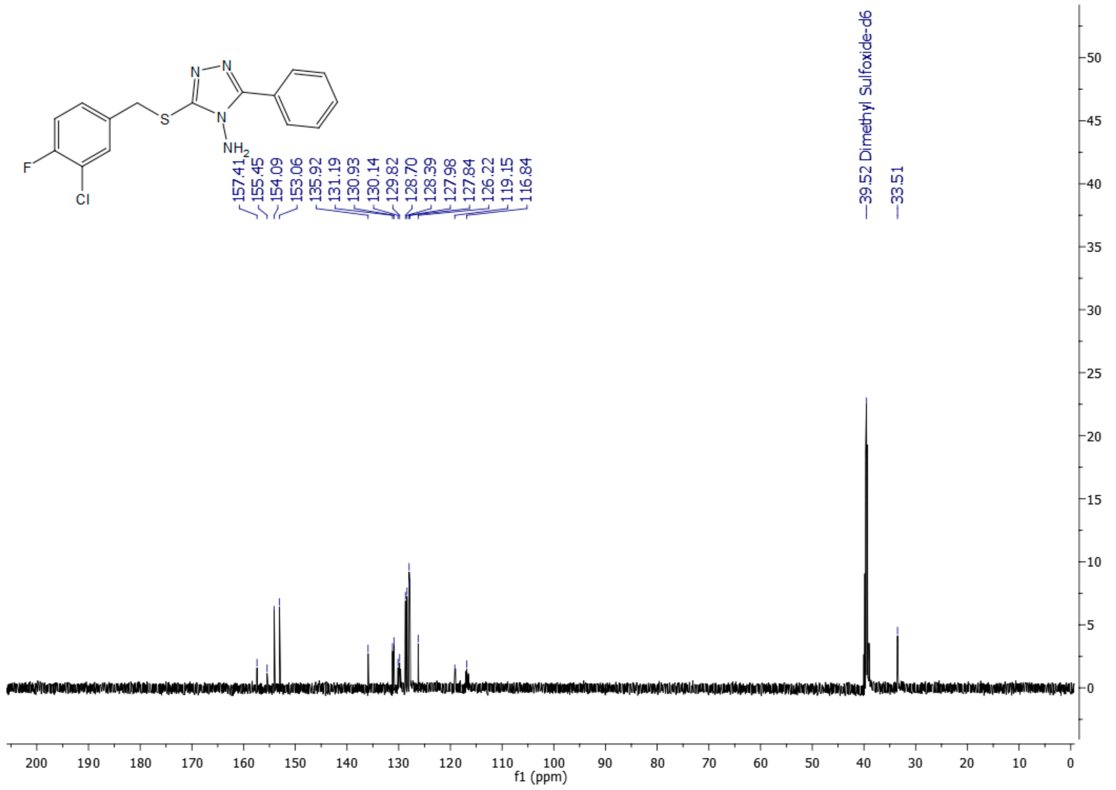

Figure S22: ^{13}C-NMR (DMSO-d_6) spectrum of 3-(3-Chloro-4-fluorobenzylthio)-5-phenyl-4H-1,2,4-triazol-4-amine (**3d**)

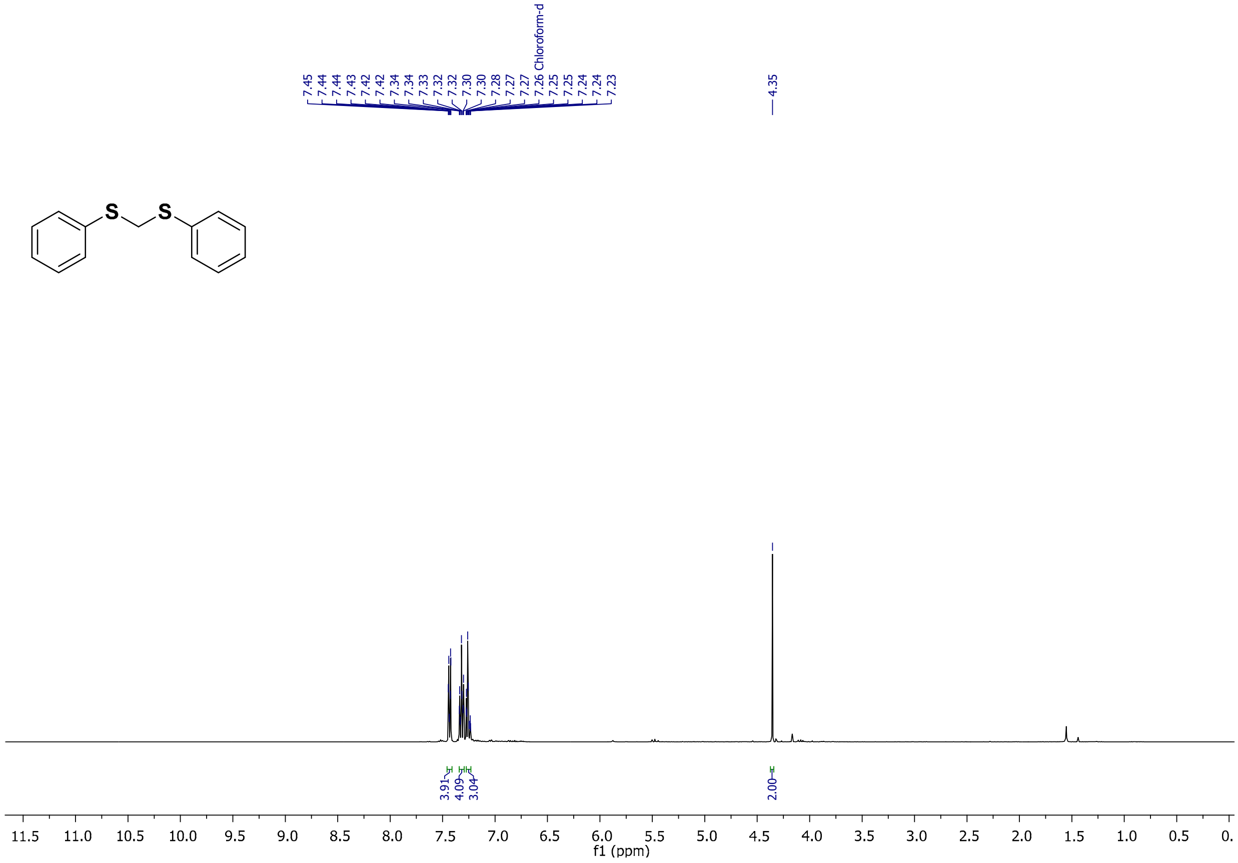

Figure S23: ^{1}H-NMR ($CDCl_3$) spectrum of phenylsulfanylmethylsulfanylbenzene (**4b**)

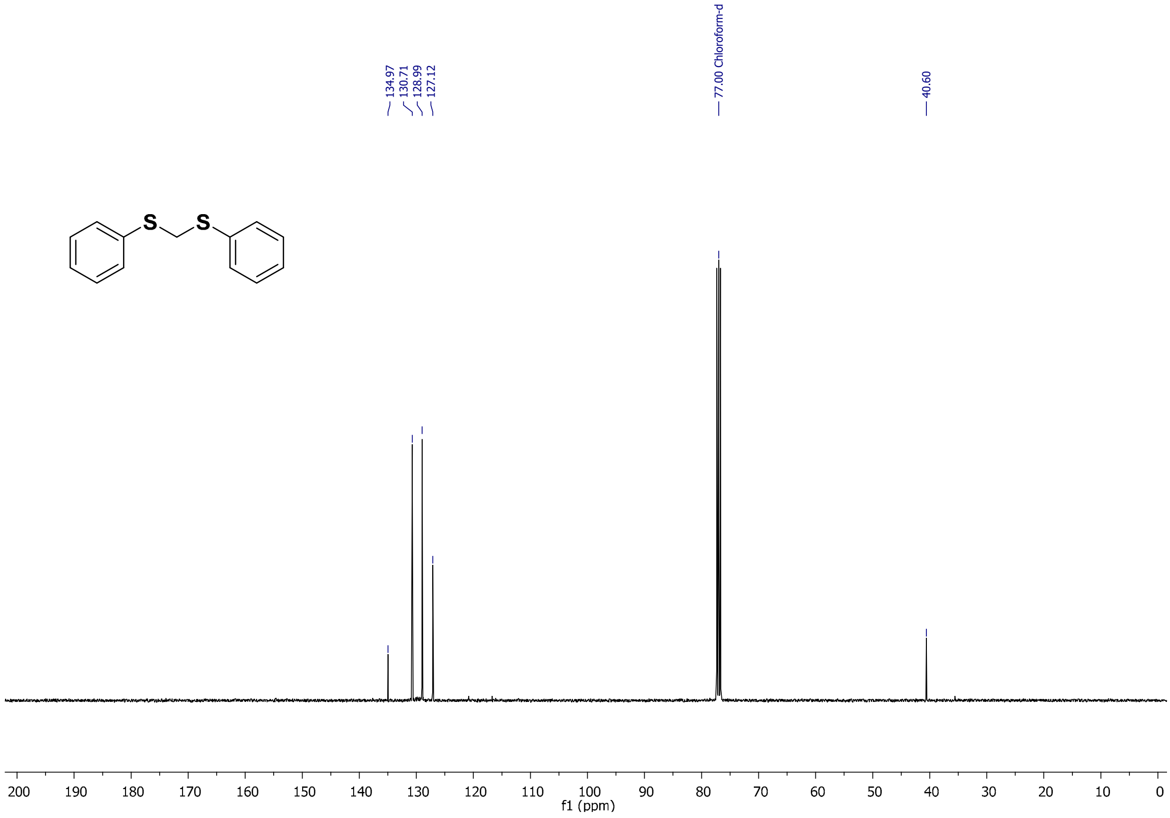

Figure S24: ^{13}C-NMR ($CDCl_3$) spectrum of phenylsulfanylmethylsulfanylbenzene (**4b**)

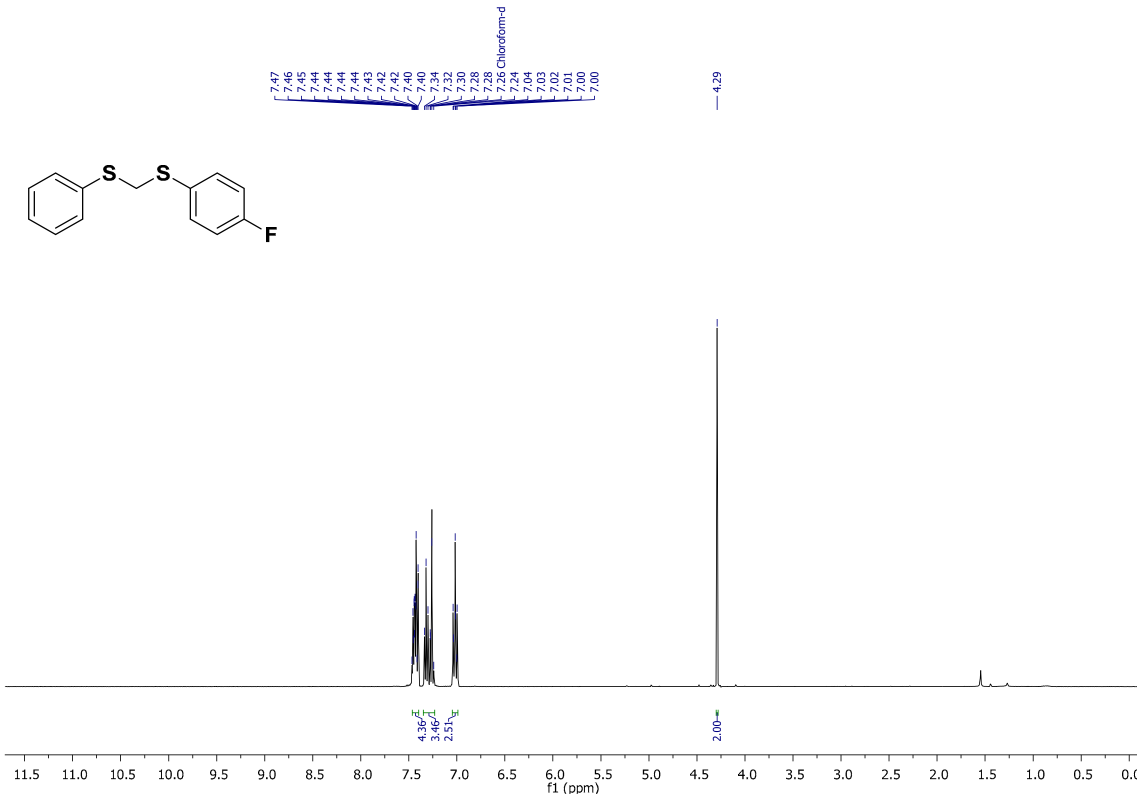

Figure S25: ^{1}H-NMR ($CDCl_3$) spectrum of 1-fluoro-4-{[(phenylsulfanyl)methyl]sulfanyl}benzene (**4c**)

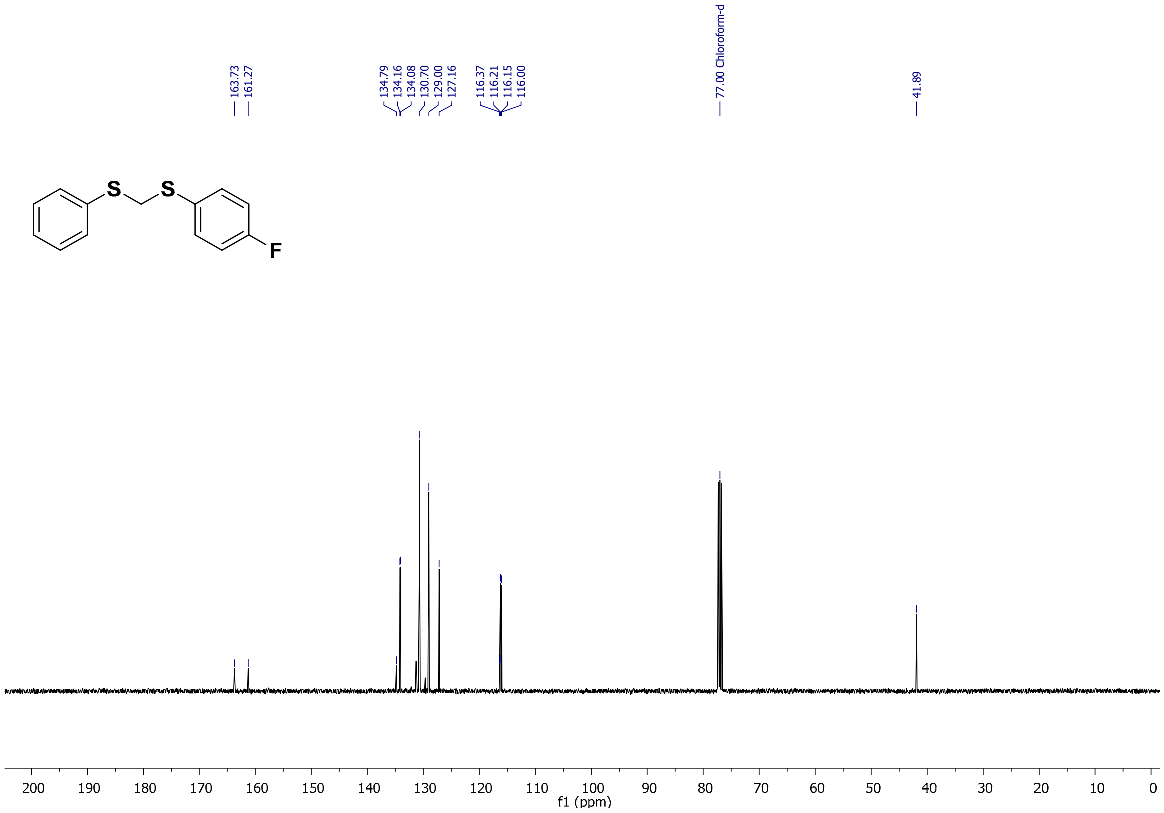

Figure S26: ^{13}C-NMR ($CDCl_3$) spectrum of 1-Fluoro-4-{[(phenylsulfanyl)methyl]sulfanyl}benzene (**4c**)

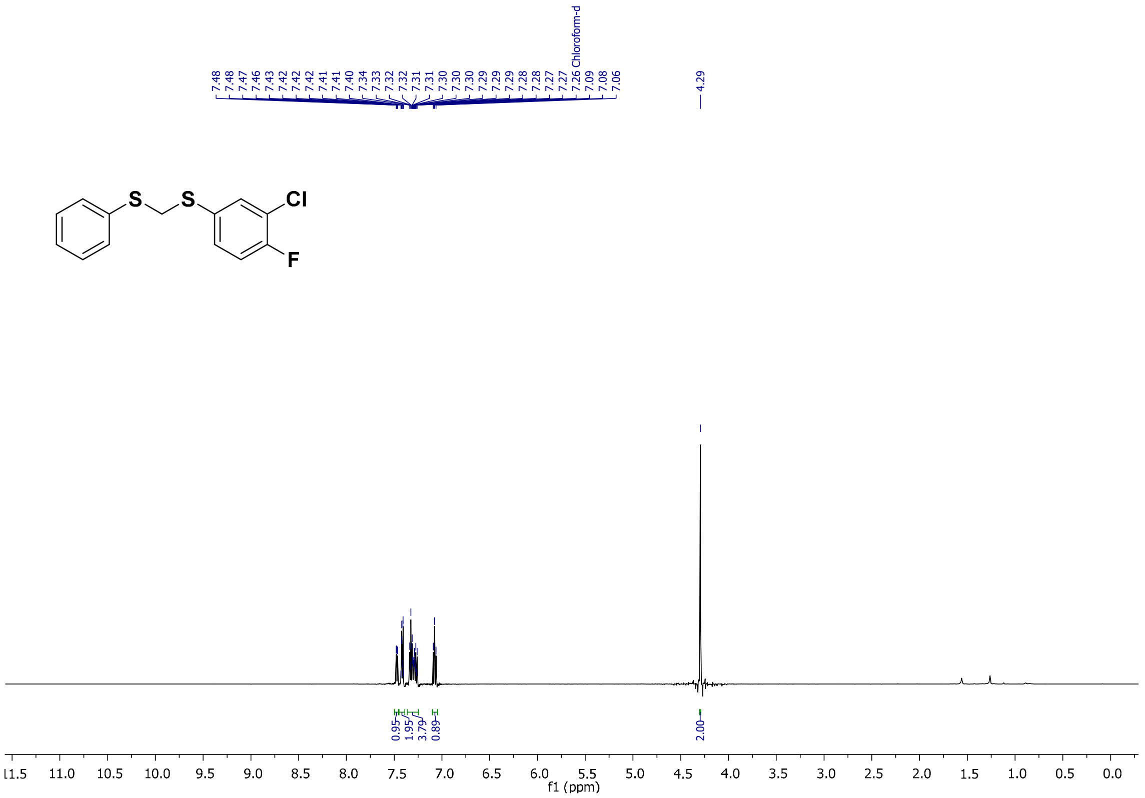

Figure S27: ^{1}H-NMR ($CDCl_3$) spectrum of 2-chloro-1-fluoro-4-{[(phenylsulfanyl)methyl]sulfanyl}benzene (**4d**)

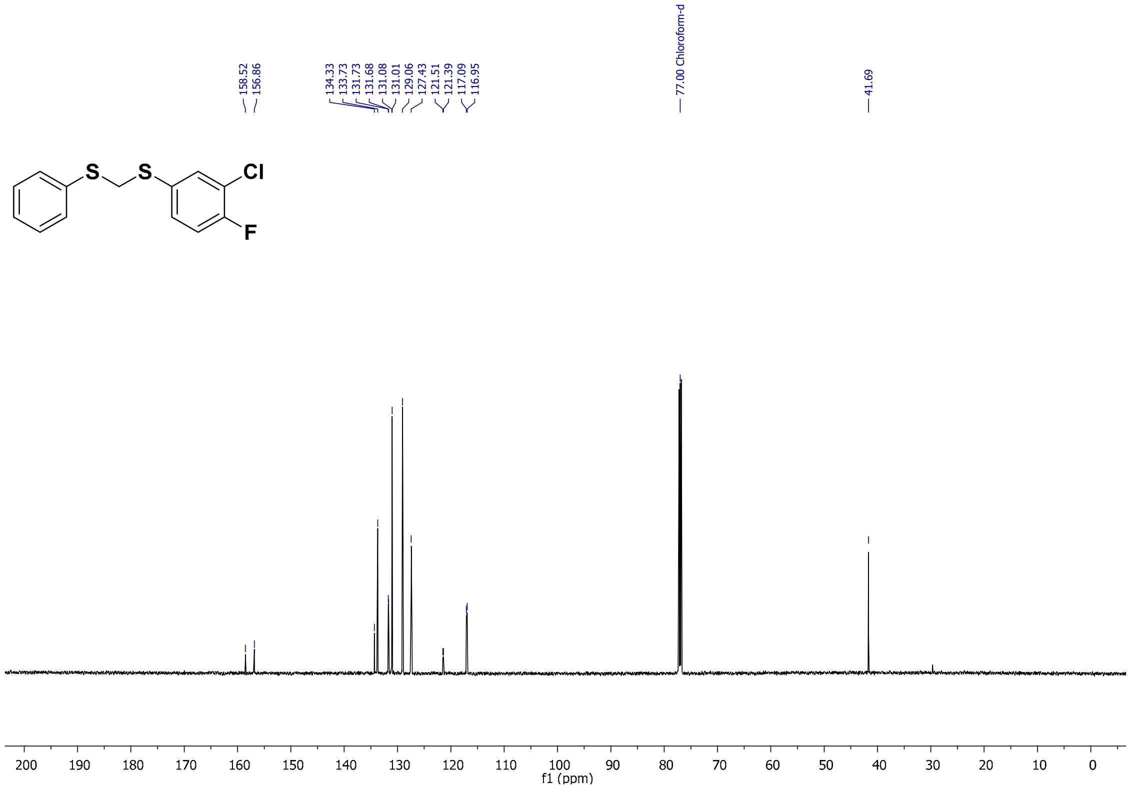

Figure S28: ^{13}C-NMR ($CDCl_3$) spectrum of 2-chloro-1-fluoro-4-{[(phenylsulfanyl)methyl]sulfanyl}benzene (**4d**)

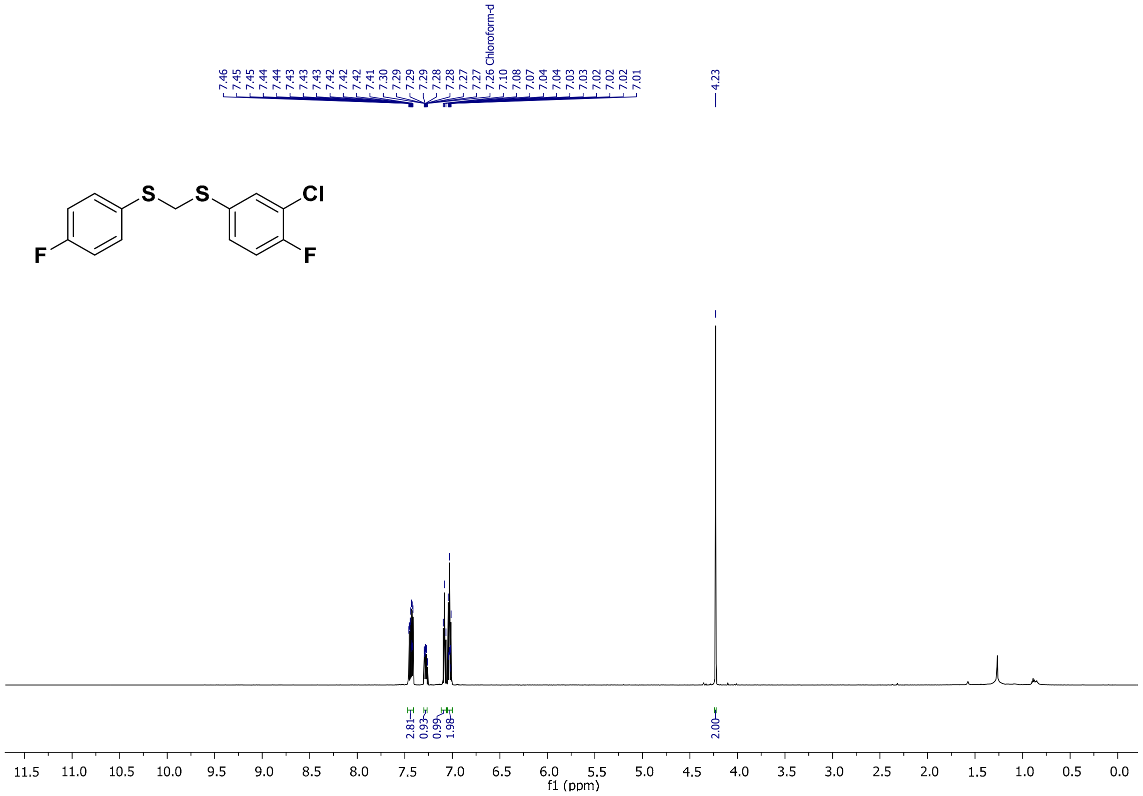

Figure S29: ^{1}H-NMR ($CDCl_3$) spectrum of 2-chloro-1-fluoro-4-(([(4-fluorophenyl)sulfanyl]methyl)sulfanyl) benzene (**4e**)

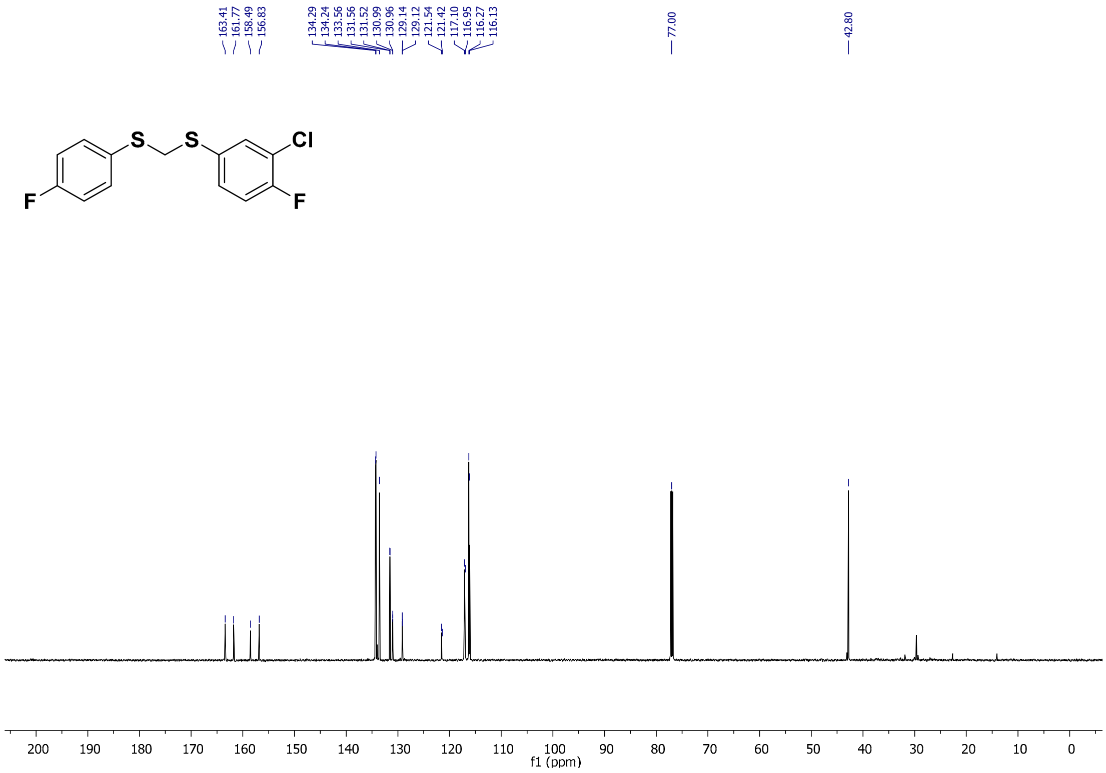

Figure S30: ^{13}C-NMR ($CDCl_3$) spectrum of 2-chloro-1-fluoro-4-(([(4-fluorophenyl)sulfanyl]methyl)sulfanyl) benzene (**4e**)

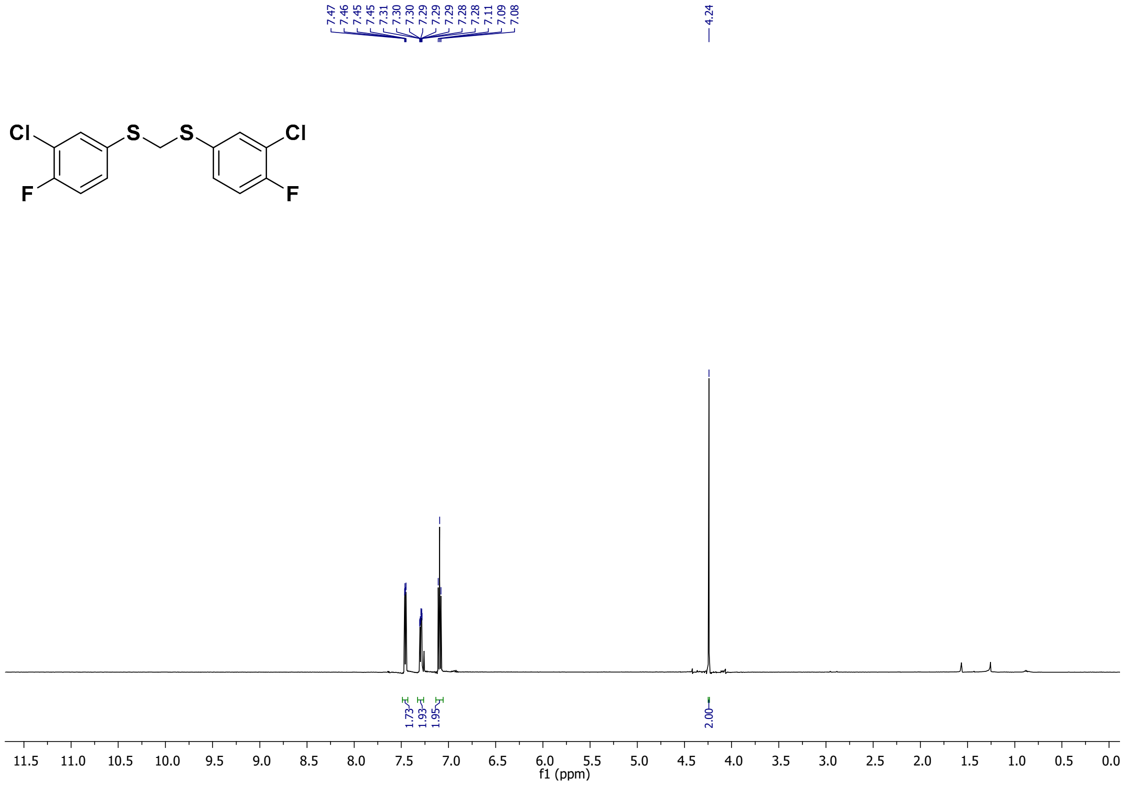

Figure S31: ^{1}H-NMR ($CDCl_3$) spectrum of 2-chloro-4-[(3-chloro-4-fluorophenyl)sulfanylmethylsulfanyl]-1-fluorobenzene (**4f**)

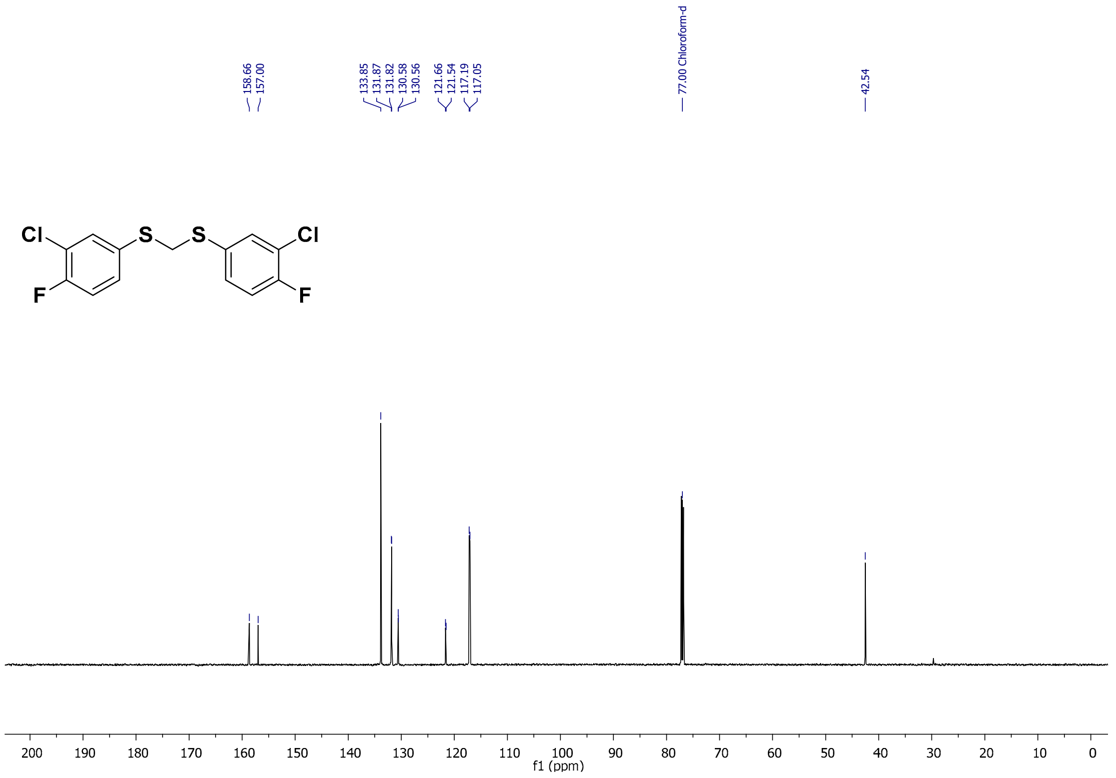

Figure S32: ^{13}C-NMR ($CDCl_3$) spectrum of 2-Chloro-4-[(3-chloro-4-fluorophenyl)sulfanylmethylsulfanyl]-1-fluorobenzene (**4f**)

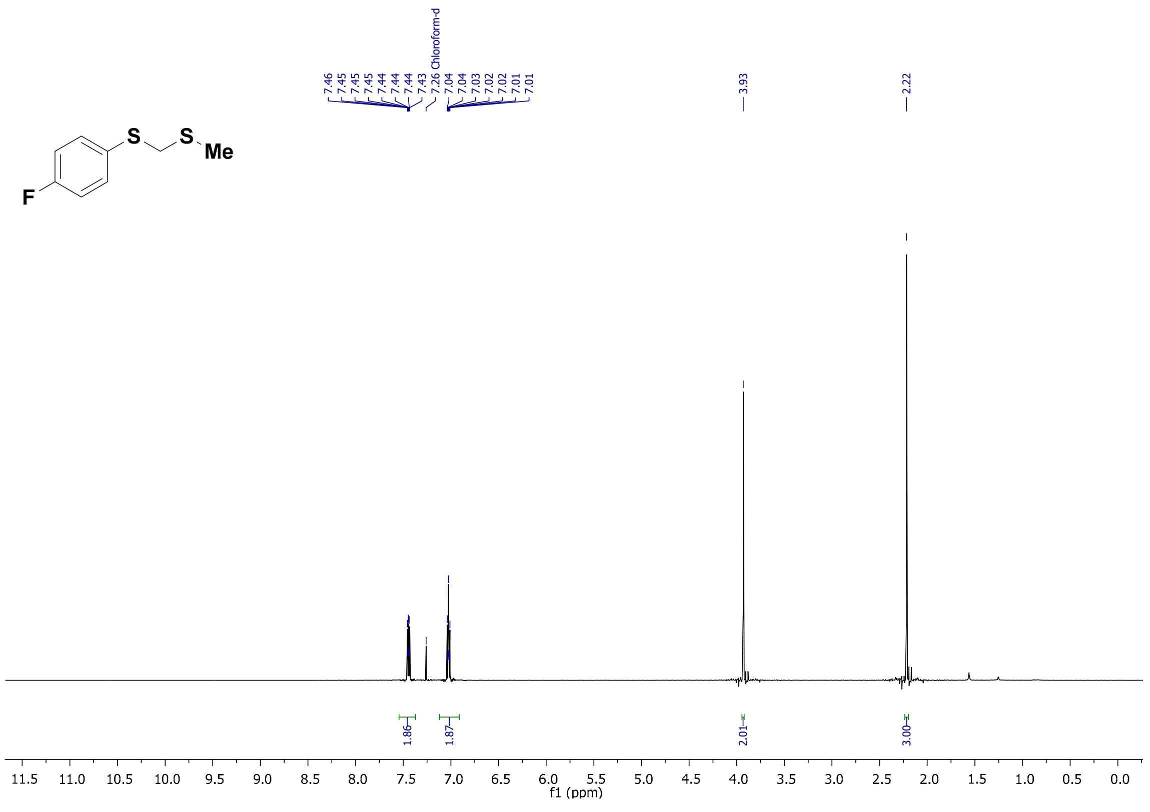

Figure S33: ^{1}H-NMR ($CDCl_3$) spectrum of 1-fluoro-4-{[(methylsulfanyl)methyl]sulfanyl}benzene **(4g)**

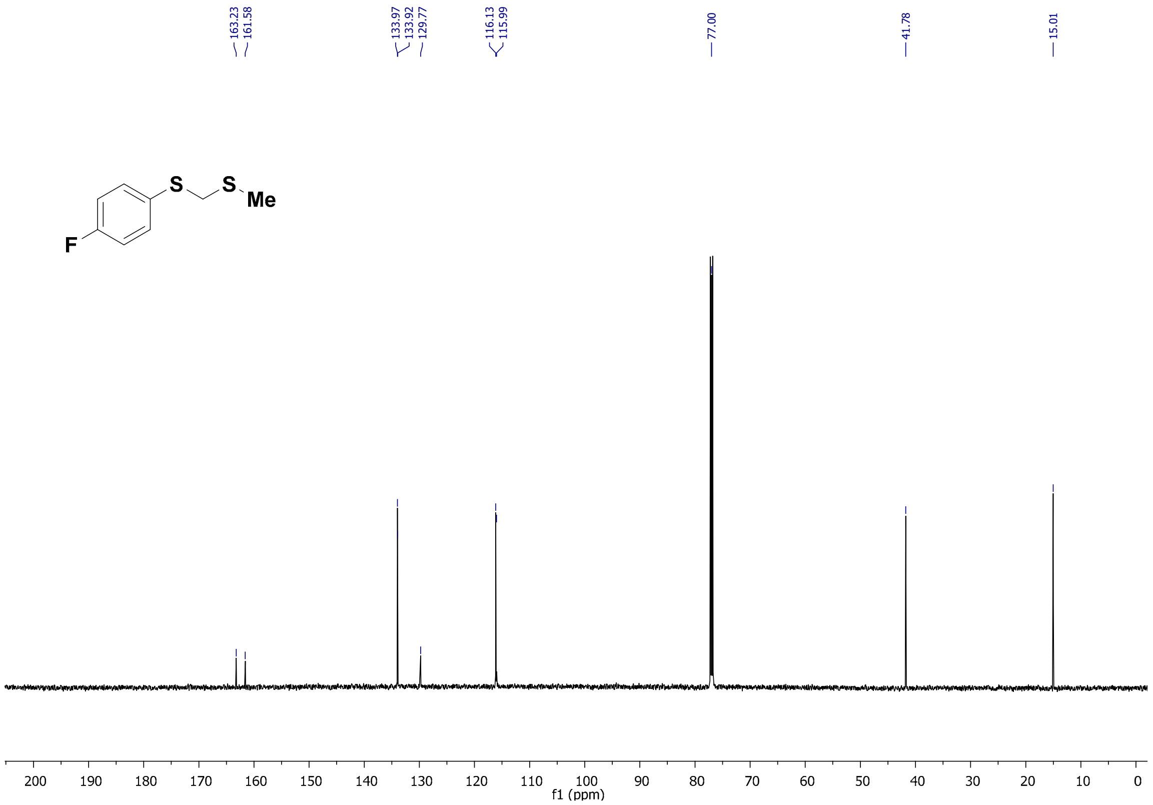

Figure S34: ^{13}C-NMR ($CDCl_3$) spectrum of 1-fluoro-4-{[(methylsulfanyl)methyl]sulfanyl}benzene **(4g)**

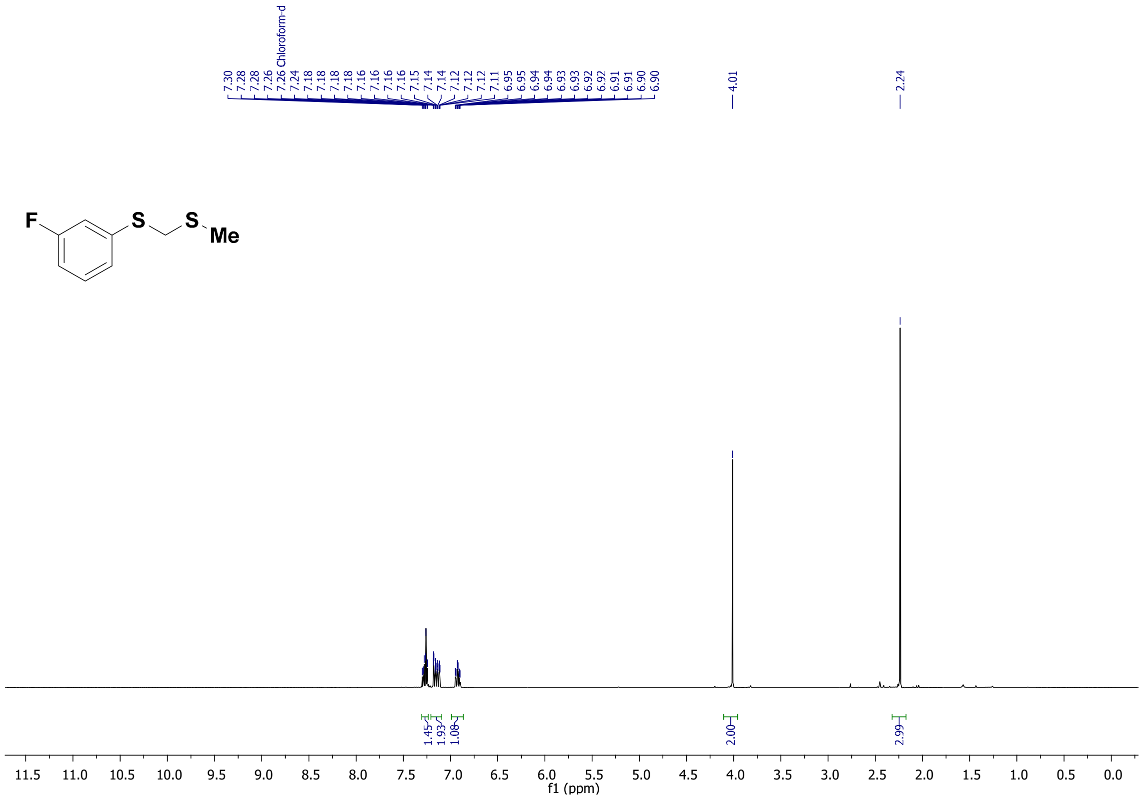

Figure S35: ^{1}H-NMR ($CDCl_3$) spectrum of 1-fluoro-3-{[(methylsulfanyl)methyl]sulfanyl}benzene (**4h**)

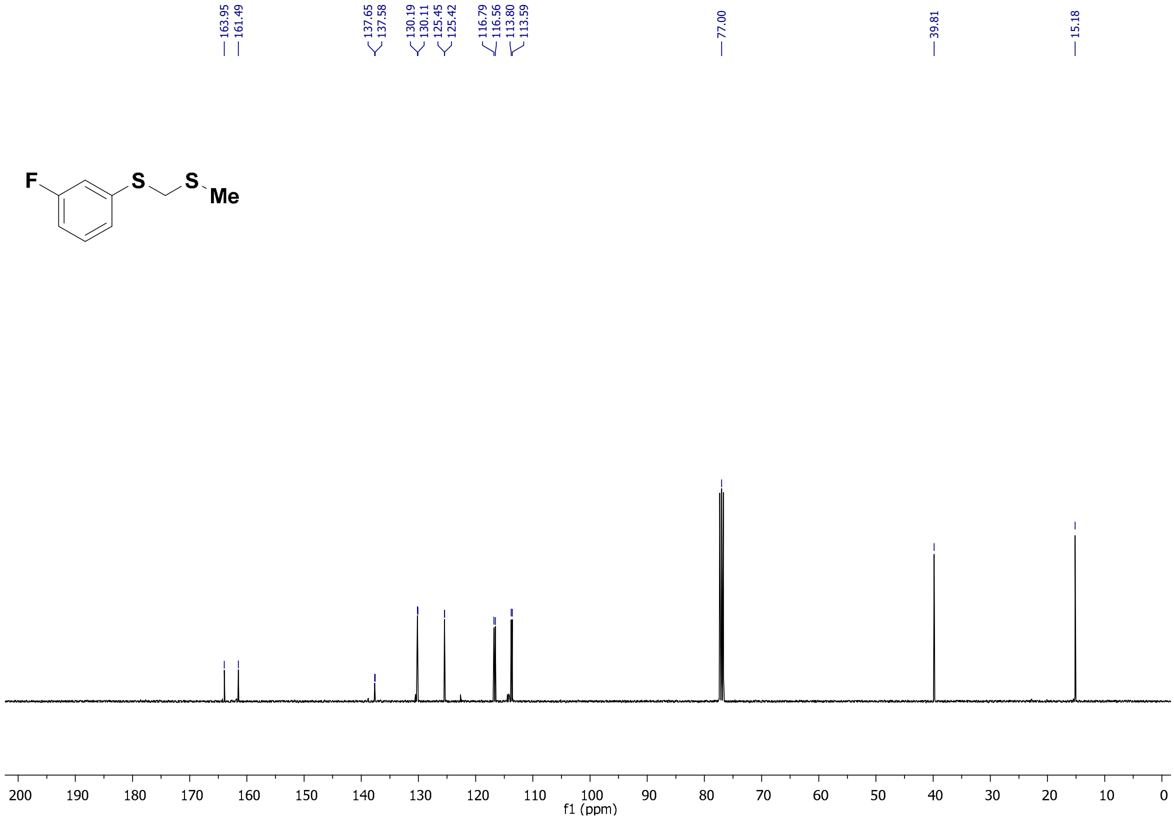

Figure S36: ^{13}C-NMR ($CDCl_3$) spectrum of 1-fluoro-3-{[(methylsulfanyl)methyl]sulfanyl}benzene (**4h**)

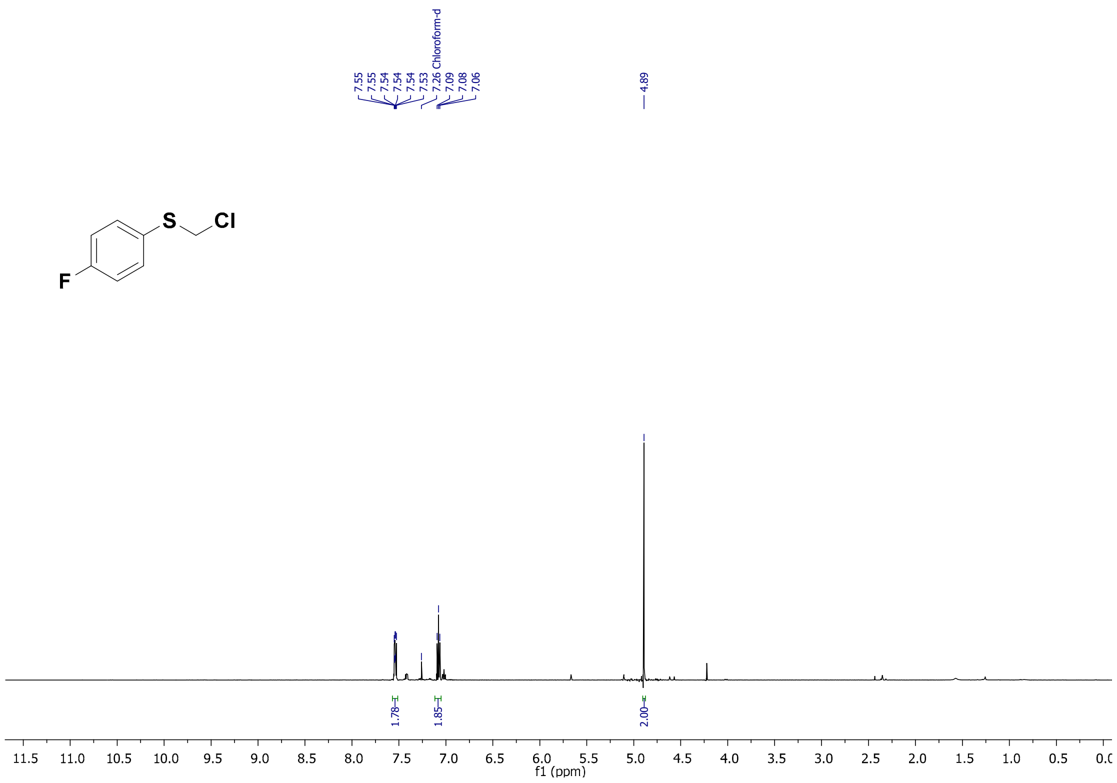

Figure S37: ^{1}H-NMR ($CDCl_3$) spectrum of 1-[(chloromethyl)sulfanyl]-4-fluorobenzene (**14c**)

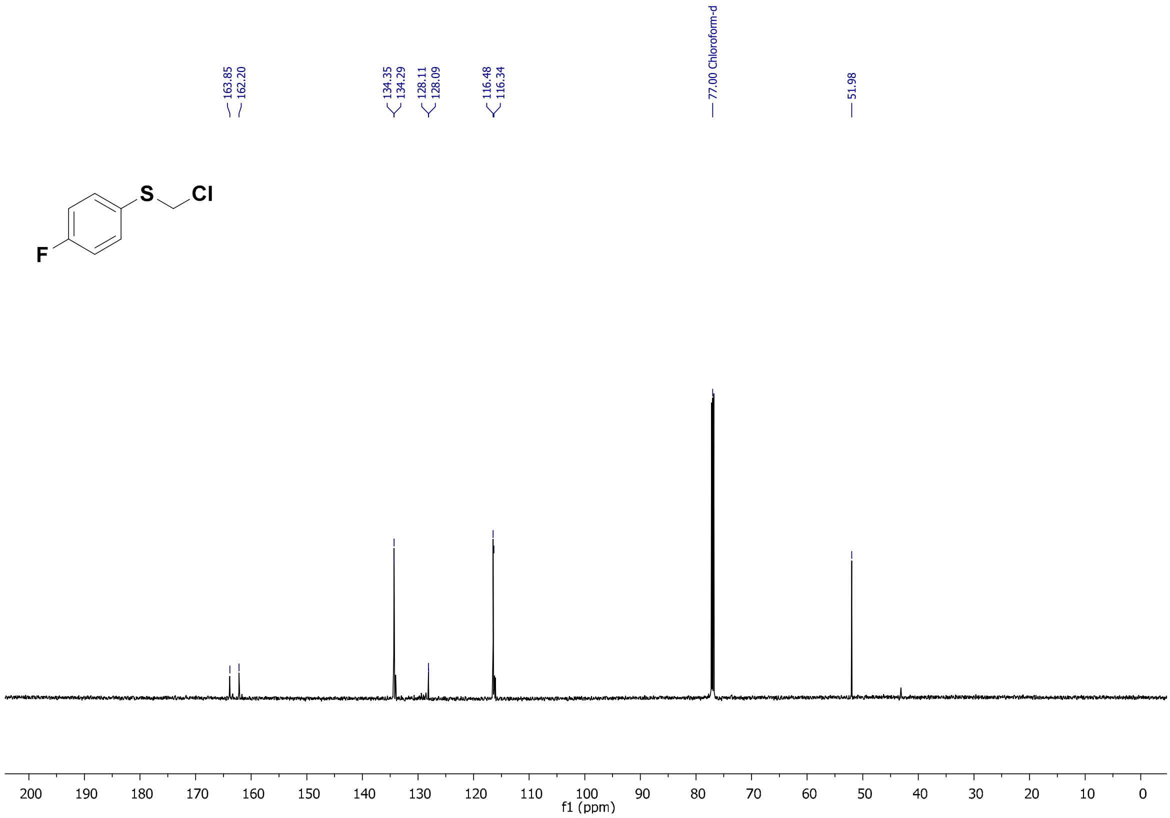

Figure S38: ^{13}C-NMR ($CDCl_3$) spectrum of 1-[(chloromethyl)sulfanyl]-4-fluorobenzene (**14c**)
